# Nudging attitudes toward IT innovations by information provision that serves as a reminder of familial support

**DOI:** 10.1371/journal.pone.0282077

**Published:** 2023-02-24

**Authors:** Hidenori Komatsu, Hiromi Kubota, Nobuyuki Tanaka

**Affiliations:** 1 Grid Innovation Research Laboratory, Central Research Institute of Electric Power Industry, Yokosuka-shi, Kanagawa, Japan; 2 Sustainable System Research Laboratory, Central Research Institute of Electric Power Industry, Abiko-shi, Chiba, Japan; Robert Gordon University, UNITED KINGDOM

## Abstract

There is concern among the general public that information technology (IT) innovations may make existing jobs redundant. This may be perceived to pose a greater problem to future generations because new technologies, not limited to IT innovations, will be sophisticated in the future. Our previous work revealed that messages reminding people of familial support as a nudge can moderate risk-averse attitudes toward risks that are perceived to threaten future generations, which could be effective for other kinds of risks. Therefore, we conducted a randomized controlled trial to examine the message effects for information provision on IT innovations. The study was conducted via an online questionnaire survey in January 2020, before the COVID-19 pandemic, and more than 3,200 samples were collected from respondents aged 20 years or older living in Japan. The treatment groups received basic information supplemented with additional text or additional text and an illustration that highlighted IT innovations as support from previous generations. The control group received only the basic textual information. The effects of the intervention were evaluated by comparing changes in average subjective assessment of IT in the treatment groups with those in the control group. The intervention effect was statistically significant, and the sense of familial support after receiving the intervention messages was significantly increased in the treatment group that viewed the illustration compared with the control group. Additionally, we discuss how each component of the HEXACO personality traits influences responses to the intervention messages. Through a series of surveys, we demonstrated the potential of our framework for a wide variety of applications involving information provision perceived to involve future generations.

## Introduction

New technologies tend to be welcomed with caution, and people perceive the risks as being high. For example, new information technology (IT), including artificial intelligence (AI) or machine learning, has attracted much media attention. The main narrative is that IT innovations will make many people’s jobs redundant and will lead to mass job losses [1–3]. One possible reason for the prevalence of this narrative is the novelty of the technologies. Unknown risks are perceived to be higher than known risks [4–6], and new, unfamiliar technologies pose unknown risks. Another possible reason is the effect on future generations. Risks are also perceived to be higher if they are expected to affect future generations [4, 7]. IT innovations are already central to society and they are growing rapidly; thus, they are perceived as probably affecting future generations. Consequently, attitudes toward IT innovations are likely risk-averse.

New technologies bring benefits as well as risks, and risk-averse attitudes in the general public can affect policy decisions, even if the benefits to the society are quantitatively obvious, and this hesitancy may harm the public good [8, 9]. The conventional way to prevent this loss of public good is provision of scientifically correct information. However, this approach is not always effective because people do not make decisions based on quantitative analysis of the balance of risks and benefits [10, 11]; in other words, the rationality is bounded [12, 13].

Nudge theory, in which behavioral change for public good is gently prompted, has been proposed in behavioral economics as a type of intervention that considers irrational human decision making [14, 15]. Among the many applications, one popular application is a messaging approach that uses social norms to encourage environmental protection. For example, providing information on how much other people are conserving energy is effective in promoting energy conservation [16]. Similarly, reusing towels in hotels can be promoted by informing guests that the majority of other guests are reusing towels [17, 18]. Healthcare is also a popular application for nudge approaches [19, 20], including using social norms as well as framing, which switches the presentation of intervention outcomes, usually from gains to losses [21, 22]. Despite a series of successful nudge applications, there are also known limitations. The target topics for messaging matter for the intervention effect sizes, as seen in the ineffective loss framing for promoting compliance with guidance during the COVID-19 pandemic [23]. Furthermore, ill-designed nudges can even backfire [24]. Ethical considerations have also been raised [25], where changing defaults have been particularly discussed in terms of transparency [26, 27].

The successful nudging applications are categorized phenomenologically [28], and the effects have been identified; however, the processes for designing nudges have relied on trial and error, and thus there is no consistent meta-theory. In parallel with behavioral economics, evolutionary psychology has been used to interpret decision making that has traditionally been regarded as irrational [29]. An increasing number of studies have sought to explain the irrational responses to risk as biological adaptations [30–34]. This approach may help to establish a consistent theory to design information provision as nudges considering intuitive human responses based on evolution [29, 35].

We proposed an approach to designing nudges that incorporates insights from evolutionary psychology, using messages highlighting familial support to moderate risk-averse attitudes [36], which was identified using an evolutionary simulation model based on kin selection theory [37, 38]. We used our approach to design information provision on air pollution caused by industrialization. For example, the provided message was as follows: ‘Industrialization, which was promoted by our parents’ and grandparents’ generations, supports every aspect of our health and quality of everyday life by providing industrial products and infrastructure, such as roads, electricity, gas, and water. The further improvement of industrialization promoted by our generation will improve the health and quality of everyday life of future generations of children.’ The message effects of the underlined sentences in the descriptions were identified, especially when adding further illustrations describing how previous generations benefit future generations [36]. This approach of including reminders of familial support could be universal and might be applied to information provision for a wider variety of risk sources.

Thus, in this study, we identify the message effects on increasing positive attitudes toward IT innovations. We use the same framework as in our previous survey for air pollution caused by industrialization, in which the intervention effects were measured via a randomized controlled trial (RCT). One traditional model for understanding the attitudes toward technology is the technology acceptance model (TAM) and its variations, which mainly assume that perceived usefulness and perceived ease-of-use affect acceptance [39, 40]. In contrast, our study investigates a new approach of stimulating a sense of familial support to promote positive attitudes, which has not been considered in TAM studies.

The effects are evaluated by segment, including basic segments, such as sex and age. A panel analysis is also performed to identify how each respondent’s attributes contribute to the effects, including personality traits. Especially in relation to personality traits, decision making that is perceived to benefit future generations relies on altruism. We identified that altruism measured as one of Big Five personality traits showed a significant contribution to the effects of similar messages [36]. Thus, in the panel analysis and in analysis for the pre-intervention attitudes, we introduced the HEXACO model [41, 42] which can be viewed as an expanded version of the Big Five model [43], to examine a broader range of personality effects.

The remainder of this paper is organized as follows. In Section 2, we describe the settings of the experiments. In Sections 3 and 4 we describe and discuss the results of the experiments, and in Section 5 we provide concluding remarks, including future work.

## Materials and methods

### Survey overview

We used a survey company that obtains samples from registered respondents. The company disseminated the online questionnaires that we designed for the experiment and the entire survey process was conducted online. We performed an RCT to identify the effects of our nudge message on attitudes toward IT innovation risks compared with simpler messages. The survey company obtained written informed consent from the respondents on our behalf. The questionnaire survey was anonymized, did not retrieve personal information, did not use samples taken from human bodies, and assumed no psychological distress of the respondents. The surveys were approved by the ethics committee of the Central Research Institute of Electric Power Industry in Japan.

The responses were collected on January 29 and 30, 2020, prior to start of the COVID-19 pandemic in Japan. Although the first case of COVID-19 was already reported on January 15, 2020, the number of cases was limited and the effects of the pandemic on this experiment were negligible during the survey term. All the respondents were over 20 years old and living in Japan. Responses were collected from an equal number of men and women. To extract only valid samples, we set two conditions that excluded unreliable samples. One condition ensures that number of children who are living with the respondents or working in paid jobs is smaller than the number of the respondents’ children. The other condition ensures that the respondents’ own income is smaller than the household income. The total number of valid samples was 3,242 and the sex ratio was 99.0, which is higher than that in the general population in Japan of 94.8 in 2019 [44].

**Fig 1:**
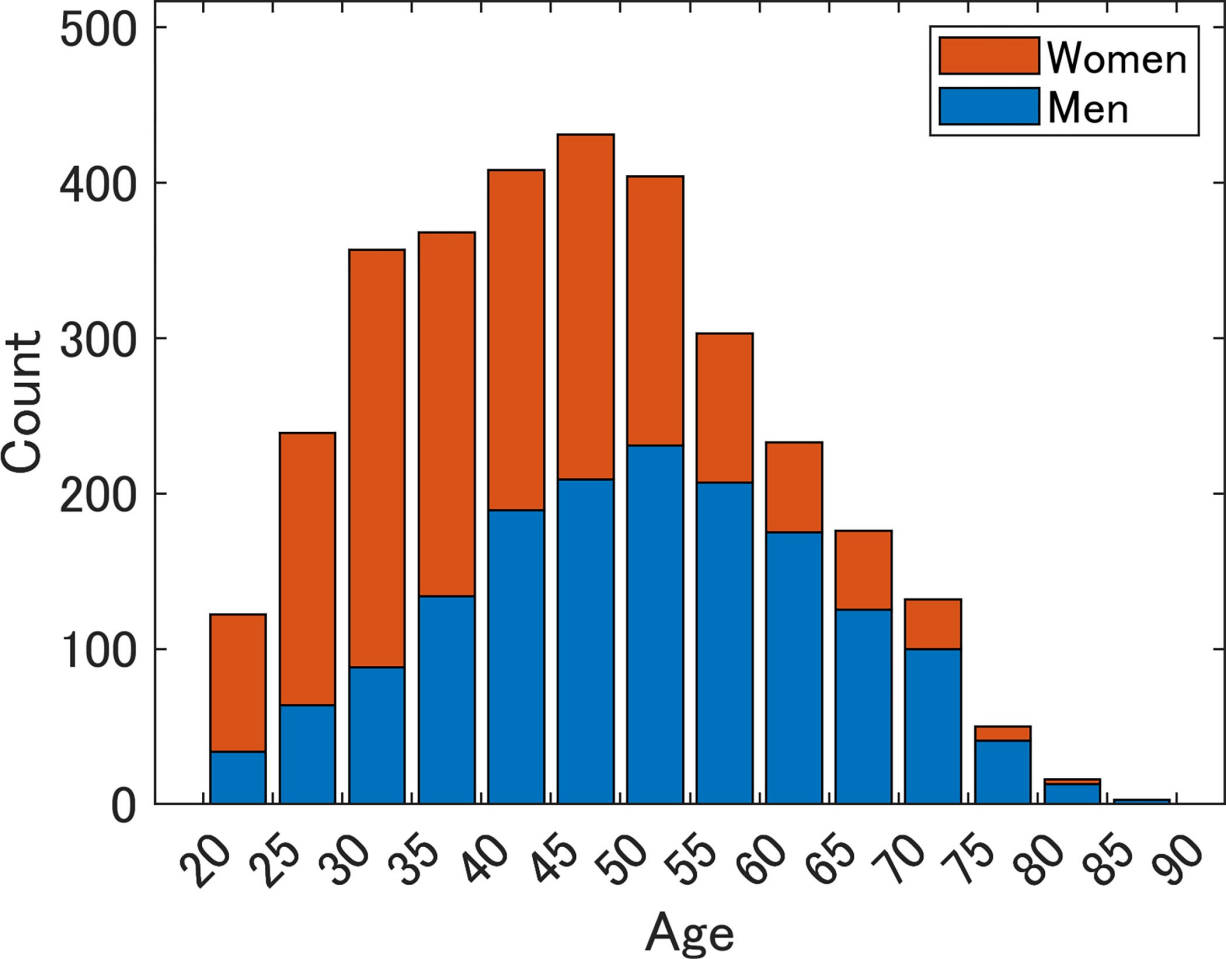
Number of respondents by age and sex.

Fig 1 shows the distribution of the respondents by age and sex. The average ages were 51.3 years for men and 41.9 years for women, resulting in an overall average age of 46.6 years (median of 46 years). A greater number of younger women were included in the samples than men. Because the estimated median age of the population of Japan in 2015 is 46.4 [45], the samples were similar to the population in terms of age.

### Survey design for intervention

**Fig 2:**
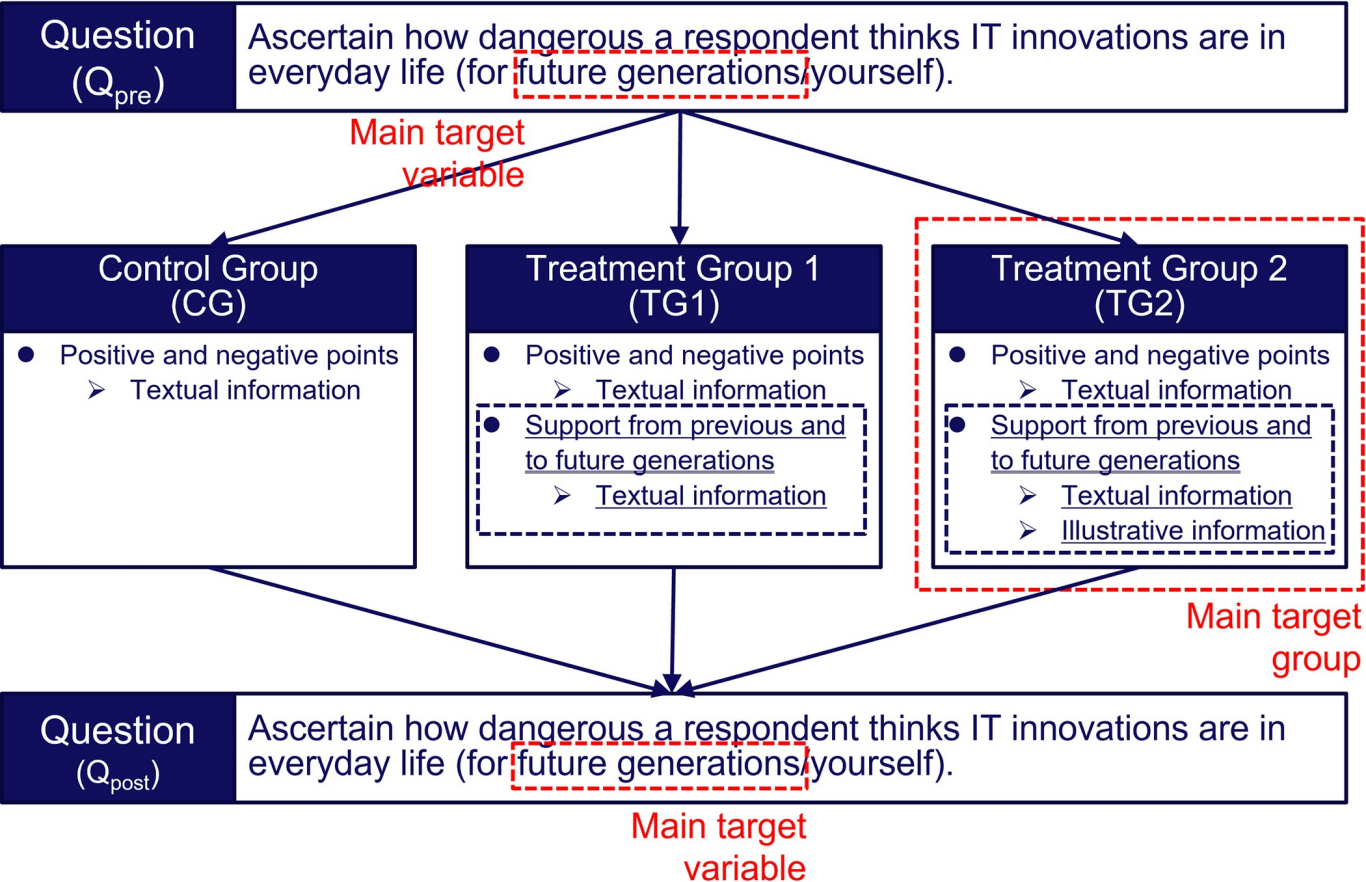
Flowchart of the experimental procedures. Black dashed lines indicate interventions. Red dashed lines indicate the main target of the interventions. This flowchart is a modified version of the experimental procedures in our previous study [36].

We conducted an RCT to ascertain whether messages reminding people of familial support could increase positive attitudes toward IT innovations, using internet-based questionnaires. Fig 2 shows the flowchart of the experimental procedures.

The experiment consisted of a question that the respondents answered twice, before (*Q*_pre_) and after (*Q*_post_) received the designed messages about IT innovations. In *Q*_pre_, the respondents were asked how dangerous or safe they think IT innovations are for future generations (Future generations) and the respondents themselves (Yourself). In *Q*_post_, the respondents were asked the same question as *Q*_pre_. For both *Q*_pre_ and *Q*_post_, the attitudes toward IT innovations were measured on a five-point Likert scale.

**Fig 3:**
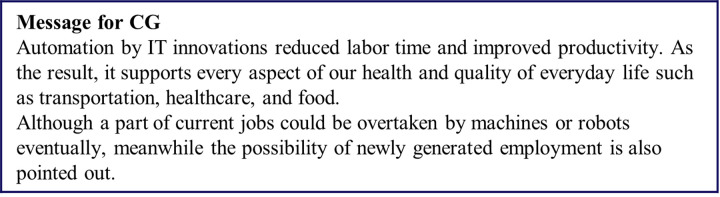
Message presented to the CG group.

**Fig 4:**
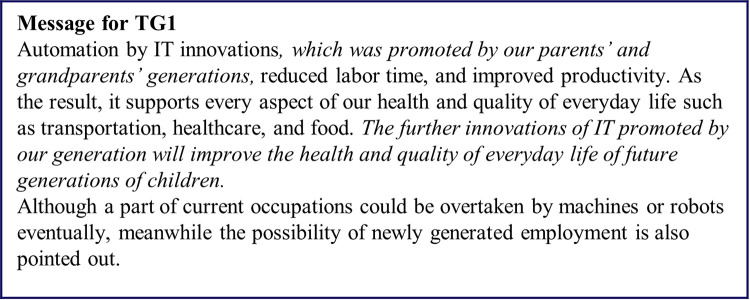
Messages presented to the TG1 group. Italic parts in the text are the intervention messages.

**Fig 5:**
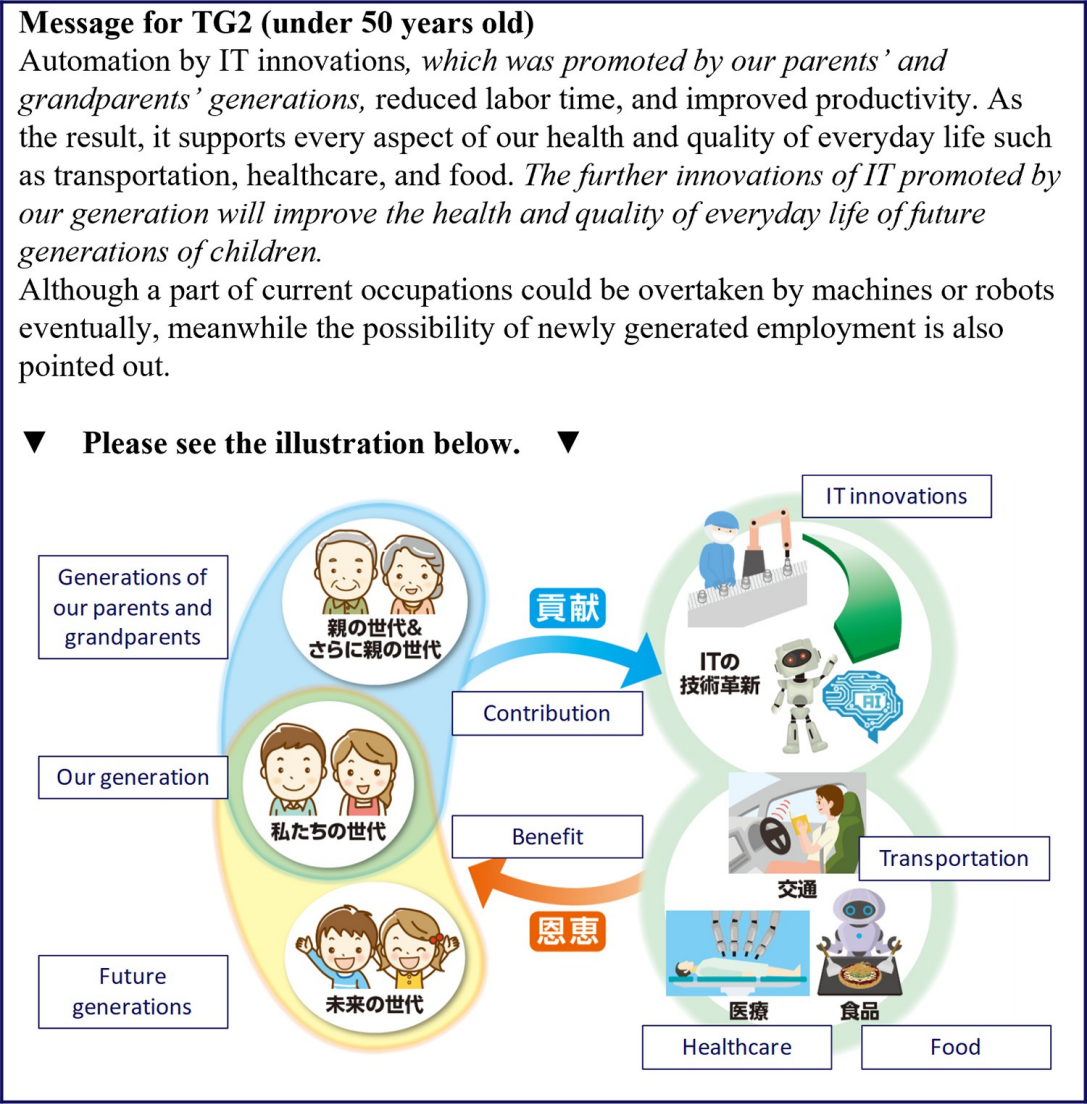
Messages presented to the TG2 group (under 50 years old). The parts of the text in italics are the intervention messages.

**Fig 6:**
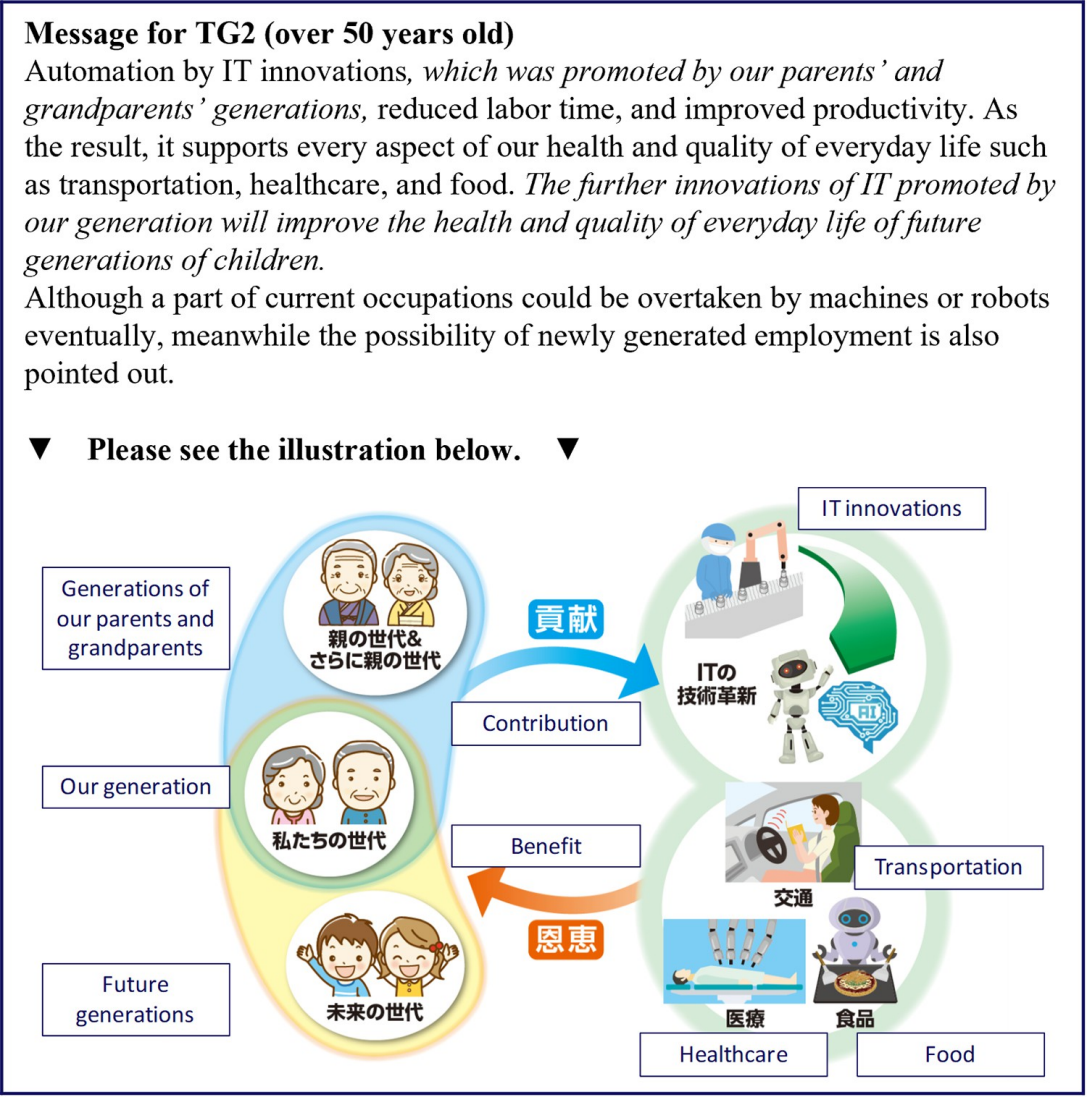
Messages presented to the TG2 group (over 50 years old). The parts of the text in italics are the intervention messages.

Between *Q*_pre_ and *Q*_post_, we used an information provision part as an intervention, where the simplest message was given to the control group (CG) and the two nudging messages were given to the treatment groups (treatment groups 1 and 2; TG1 and TG2). The respondents were randomly assigned to either CG, TG1, or TG2. The messages were designed so that the information got richer from CG to TG2 and the effect of each piece of additional information should be separable (Table 1, Figs 3–6).

**Table 1:** Group definitions in the interventions.

Information provided		Presentation type	CG	TG1	TG2 (main target group)
	Positive and negative points of IT innovations	Textual	✓	✓	✓
	Support from previous generations and to future generations	Textual		✓	✓
		Illustrative			✓
**Number of samples**			1,074	1,091	1,077

CG, control group receiving only information about the risks and benefits of IT innovations; TG1, treatment group 1 receiving additional information about support from previous generations and to future generations; TG2, treatment group 2 receiving an additional illustration highlighting the structure of support from previous generations and to future generations. ✓ Indicates the information was provided.

Fig 3 shows the messages for the CG, who received the simplest information, which consisted only of text. We designed this message to be as concise and fair as possible, referring to both the positive and negative aspects of recent IT innovations. Positive points highlighted that IT supports aspects of our daily life, such as transportation, healthcare, and food. Negative points highlighted the possibility that some current jobs could be automated and lost, although we also state that that new employment would eventually be generated.

Fig 4 shows the messages for TG1, who received the information that consisted only text with two additional pieces of texts related to familial support. The first piece of additional text described the benefits of previous IT innovations as products of previous generations. The second piece of additional text said that future IT innovations would support future generations, which was expected to increase the sense of familial support from previous generations by highlighting the possibility that the costs to the respondents of supporting future generations would be decreased.

Figs 5 and 6 show the messages for TG2, which is our main target group for this experiment. Although the textual message was identical to that for TG1, only TG2 received additional illustrative information. The illustration highlighted how previous generations and present generations promote IT innovations, and that the benefits are received by present and future generations, respectively. The differences between Figs 5 and 6 are the illustrations for the previous and the current generations, where the ages of the people pictured are tailored for the generation that the respondents belong to. Because many of the respondents over the age of 50 years will probably not have living parents, the people in the previous generations are wearing traditional clothes (Fig 6).

Although TG2 and TG1 were presented with essentially the same information, TG2 showed the largest intervention effects in the previous study, followed by TG1, whereas CG showed the weakest effects. The difference in effects between the two treatment groups was caused by the illustration given to TG2. TG1 received text that was complicated to interpret; thus, the additional visual information given to TG2 helped to convey the supportive relationship across generations more powerfully, which increased the sense of familial support and boosted the intervention effects compared with TG1. We expected to observe a similar effect in the present study for information provision on the IT innovations.

## Results

### Pre-intervention attitudes

**Fig 7:**
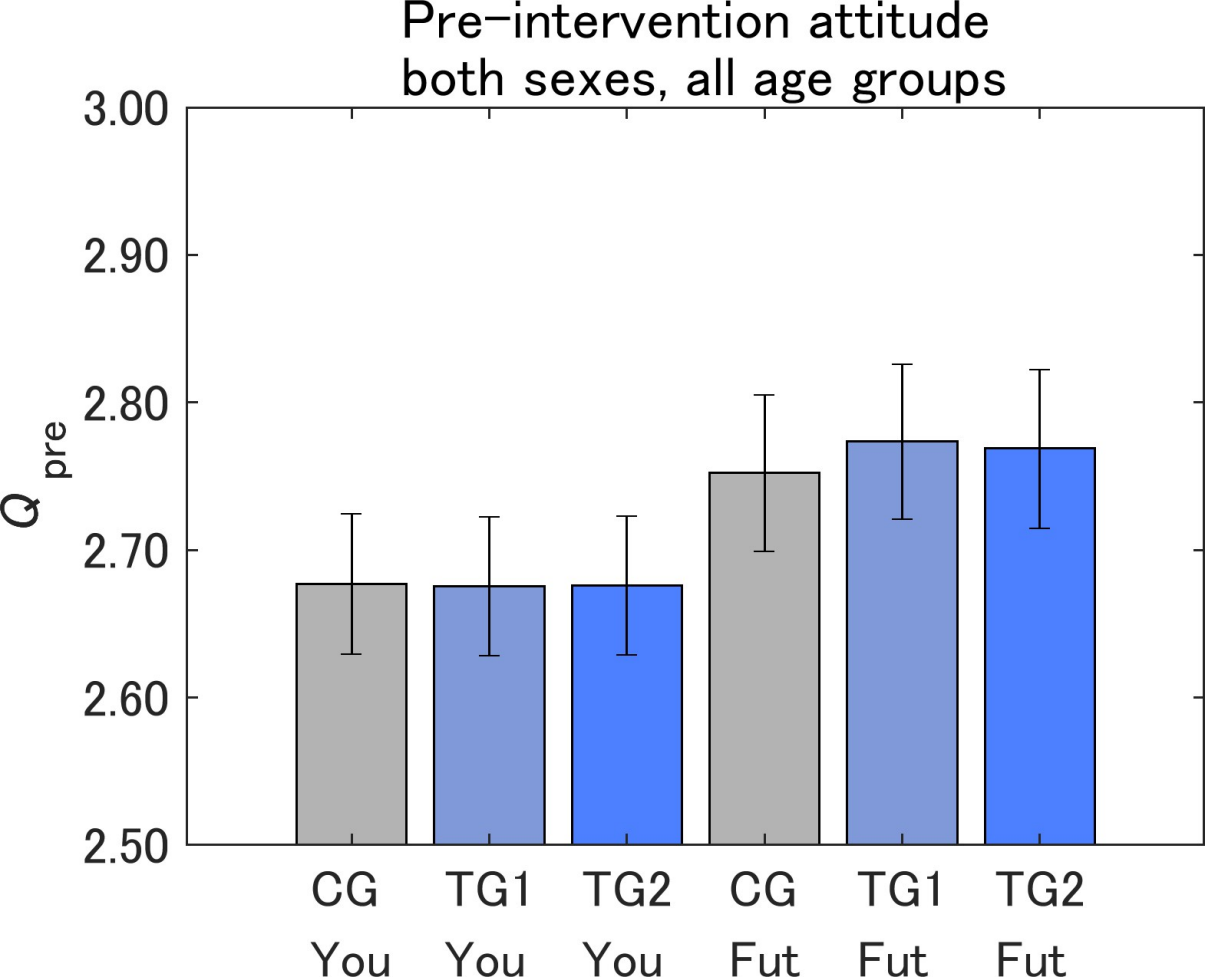
Pre-intervention attitudes toward IT innovations. The value on the vertical axis is higher the more dangerous IT innovations are perceived to be. CG, control group; TG1, treatment group 1; TG2, treatment group 2; You, Yourself; Fut, Future generations. Error bars show 95% confidence intervals.

Fig 7 shows the pre-intervention attitudes toward IT innovations (*Q*_pre_). Analysis of variance for the respondents themselves (Yourself) and future generations (Future generations) is shown in Tables 2 and 3, respectively. Comparisons of pre-intervention attitudes toward IT innovations are shown in Tables 4 and 5. The differences in the pre-intervention attitudes were statistically nonsignificant among CG, TG1, and TG2. This suggests that the samples were sufficiently randomized. Within the same message group, the pre-intervention attitudes were consistently more risk-averse for Future generations than for Yourself.

**Table 2:** One-way analysis of variance for pre-intervention attitudes toward IT innovations (*Q*_pre_) for the respondents themselves (Yourself).

Source	SS	df	MS	F	Probability > F
**Group**	0.001	2.000	0.001	0.001	0.999
**Error**	2039.931	3239.000	0.630		
**Total**	2039.932	3241.000			

SS, sum-of-squares; df, degrees of freedom; MS, mean squares; F, F ratio.

**Table 3:** One-way analysis of variance for pre-intervention attitudes toward IT innovations (*Q*_pre_) for future generations (future generations).

Source	SS	df	MS	F	Probability > F
**Group**	0.269	2.000	0.134	0.170	0.844
**Error**	2560.631	3239.000	0.791		
**Total**	2560.899	3241.000			

SS, sum-of-squares; df, degrees of freedom; MS, mean squares; F, F ratio.

**Table 4:** Comparison of pre-intervention attitudes toward IT innovations (*Q*_pre_) for the respondents themselves (Yourself).

Compared groups		Lower end of 95% confidence interval	Estimated mean	Upper end of 95% confidence interval	*p*
CG	TG1	−0.079	0.001	0.081	0.999
CG	TG2	−0.079	0.001	0.081	1.000
TG1	TG2	−0.080	0.000	0.079	1.000

**Table 5:** Comparison of pre-intervention attitudes toward IT innovations (*Q*_pre_) for future generations (future generations).

Compared groups		Lower end of 95% confidence interval	Estimated mean	Upper end of 95% confidence interval	*p*
CG	TG1	−0.111	−0.021	0.068	0.843
CG	TG2	−0.106	−0.016	0.073	0.903
TG1	TG2	−0.085	0.005	0.094	0.991

**Fig 8:**
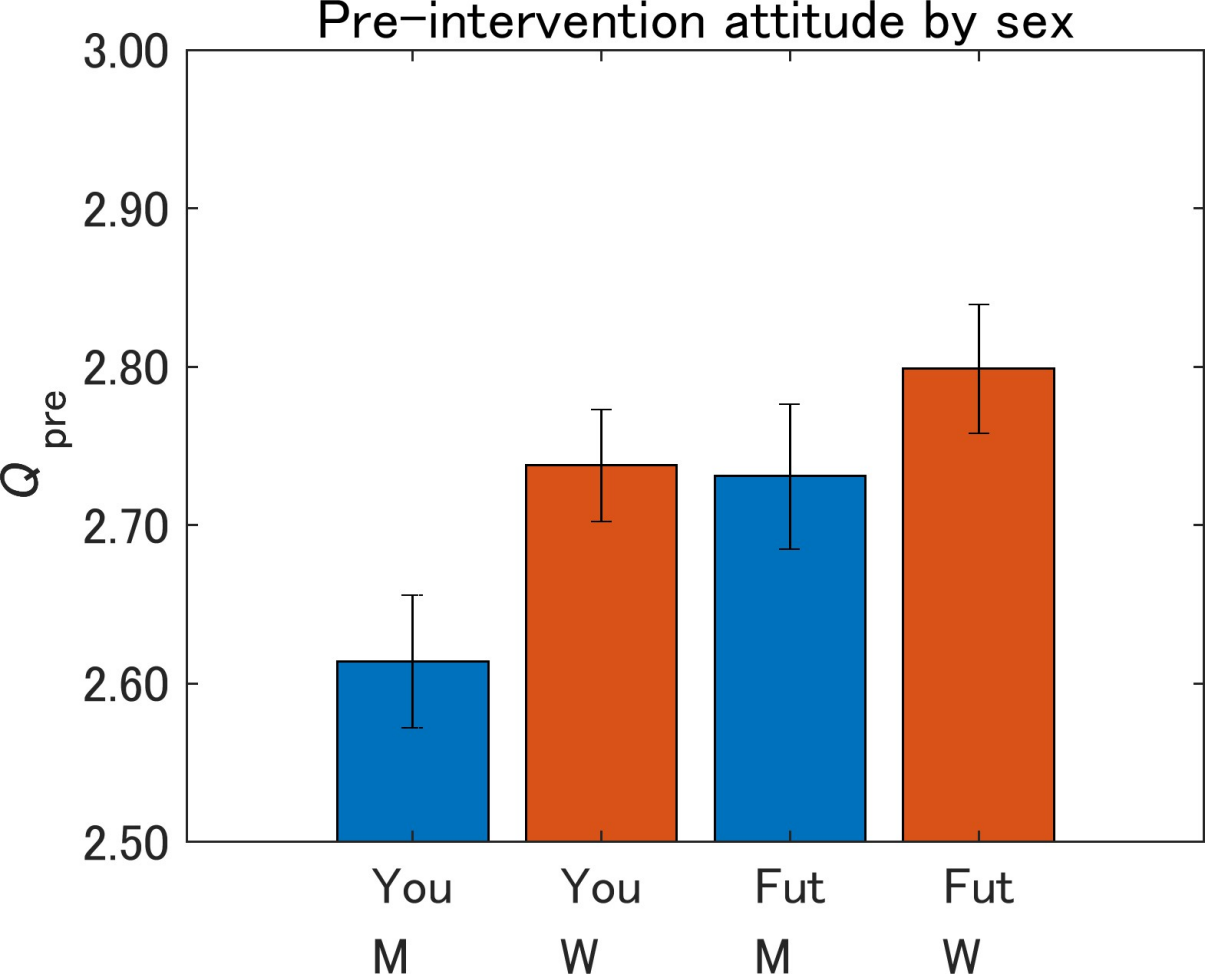
Pre-intervention attitudes toward IT innovations by sex. You, Yourself; Fut, Future generations; M, men; W, women. The value on the vertical axis is higher the more dangerous IT innovations are perceived to be. Error bars show 95% confidence intervals.

Fig 8 shows the pre-intervention attitudes toward the IT innovations by sex. For both Yourself and Future generations, women showed more risk-averse attitudes than men within the same message group. Within the same sex, the attitudes were more risk-averse for Future generations than for Yourself, which were statistically significant for both men (*p* < 0.001) and women (*p* < 0.05).

**Fig 9:**
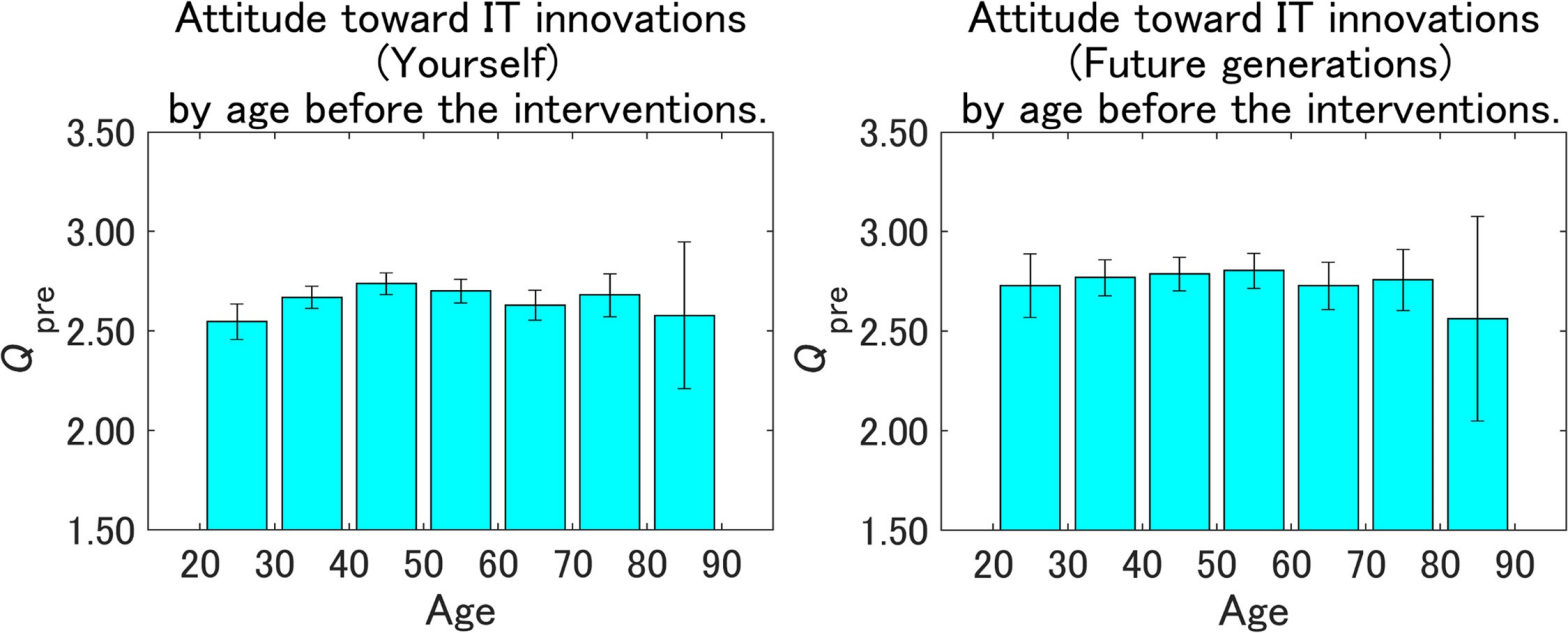
Pre-intervention attitudes toward IT innovations by age. The value on the vertical axis is higher the more dangerous IT innovations are perceived to be. Error bars show 95% confidence intervals.

Fig 9 shows the pre-intervention attitudes toward IT innovations by age. Although there were no significant differences among the age groups for Future generations, the 20–30- and 30–40-year-old groups were less risk-averse for Yourself. Because the values of *Q*_pre_ were not significantly smaller for Yourself than for Future generations within the other age groups, the less risk-averse 20–30- and 30–40-year-old groups for Yourself may be a major reason for the less risk-averse attitudes for Yourself than for Future generations shown in Fig 7. The 80–90-year-old group tended to be less risk-averse for both Future generations and Yourself, although there was no statistical significance. Thus, there was no clear linear relationship between age and the pre-intervention attitudes toward IT innovations for both Future generations and Yourself.

Table 6 shows correlation coefficients between the pre-intervention risk-averse attitudes toward IT innovations and HEXACO measures for Yourself and Future generations, using the whole responses. All the correlation coefficients were significant, where Honesty-Humility and Emotionality showed negative correlations, whereas Extraversion, Agreeableness, Conscientiousness, and Openness showed positive correlations.

**Table 6:** Correlations between pre-intervention risk-averse attitudes toward IT innovations and HEXACO measures.

	Honesty-Humility	Emotionality	Extraversion	Agreeableness	Conscientiousness	Openness
**Yourself**	***0.087	**0.056	***-0.102	***-0.087	**-0.046	***-0.080
**Future generations**	***0.069	*0.036	***-0.072	*-0.044	*-0.036	**-0.051

‘, *, **, *** difference from zero with 90%, 95%, 99%, and 99.9% confidence, respectively.

### Overall intervention effects

To evaluate the difference-in-difference (DID) [46] effect of the interventions, we defined the difference between the pre-intervention attitudes (*Q*_pre_) and the post-intervention attitudes (*Q*_post_) toward the IT innovations as *D*. We calculated the mean value of *D* for each message group, and then evaluated the DID effect by checking whether the values for TG1 and TG2 were larger than that for CG.

**Fig 10:**
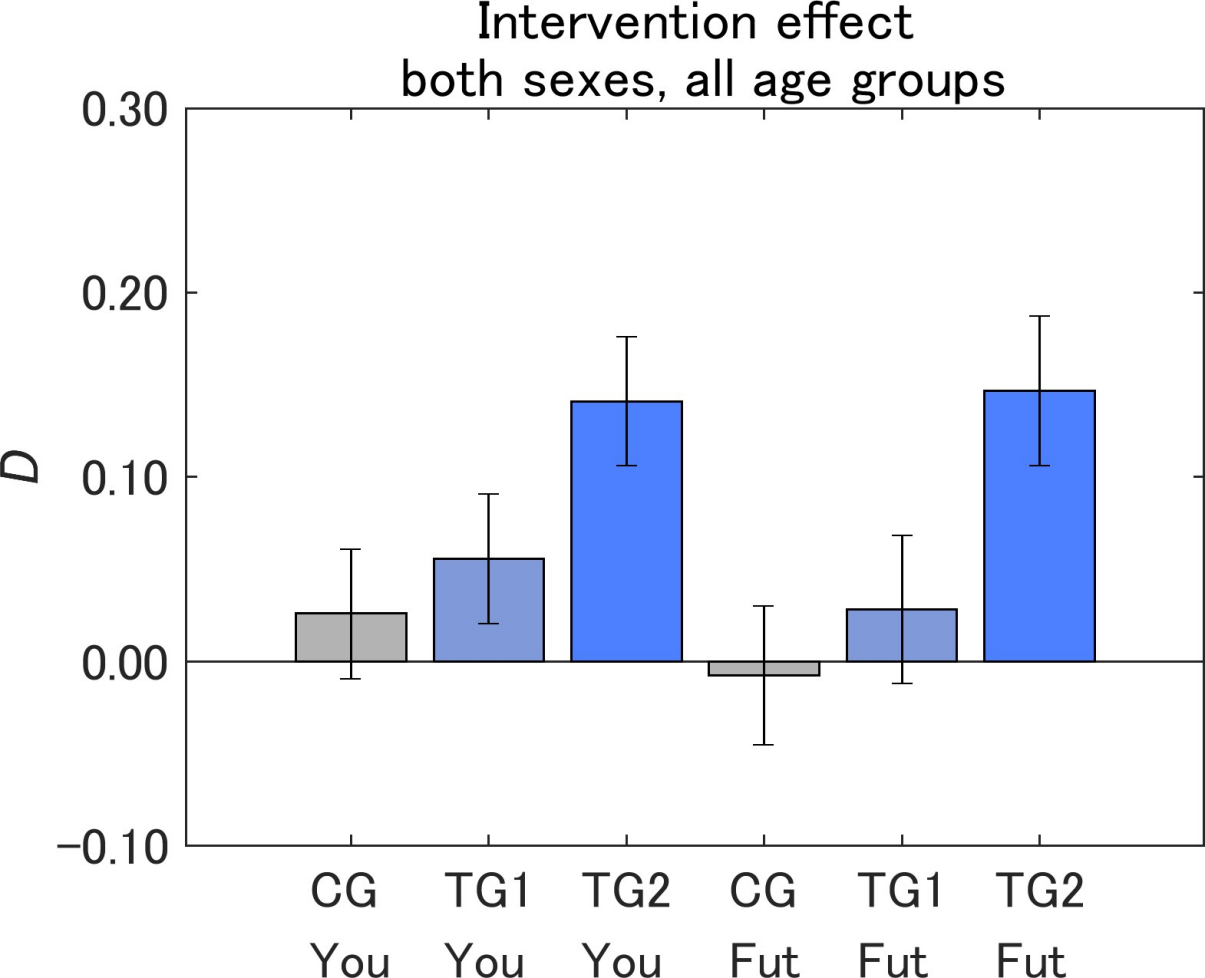
Degree of attitude change toward IT innovations (*D*) by message group. The value on the vertical axis is higher the less dangerous the perception of IT innovations became. Error bars show 95% confidence intervals.

Fig 10 shows *D* by message group. For both Yourself and Future generations, the order of the averages was TG2 > TG1 > CG. The values of *D* for CG were not significantly different from 0 for both Yourself and Future generations, suggesting that the messages for these control groups functioned neutrally, as we intended, not to alter the attitudes toward IT innovations. TG2 showed a significant increase in *D* from 0, CG, and even TG1 for both Yourself and Future generations (*p* < 0.001). Although TG1 showed an increase in *D* compared with CG, the differences were not significant for both Yourself and Future generations. The results suggest that presenting the information visually as an illustration to TG2 was effective for increasing *D* compared with CG for both Yourself and Future generations, where the insignificant intervention effects in TG1 were successfully boosted by just adding the illustration of the same messages as in TG1.

The post-intervention values of *D* and the DID effects were similar for Yourself and Future generations, although the pre-intervention attitudes were more risk-averse for Future generations than for Yourself (Fig 7). However, in our previous study on information provision about air pollution, the intervention effects were weaker for Yourself than Future generations [36]. The current study shows that our designed message can increase positive attitudes both for Future generations and Yourself on some topics, even if the pre-intervention attitudes are more risk averse for Future generations than for Yourself.

**Fig 11:**
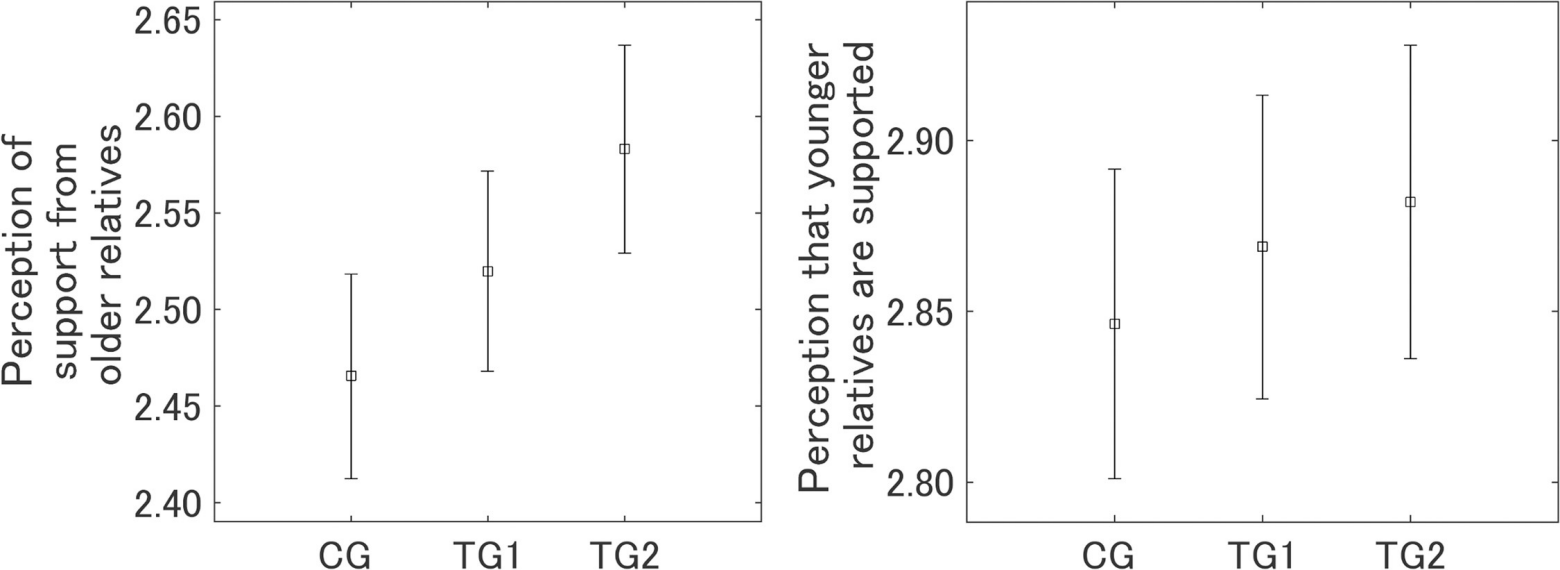
Sense of familial support on reading the designed messages. Error bars show 95% confidence intervals.

Respondents were asked how much they felt their daily life was supported by their older relatives, including parents and grandparents, on reading the designed messages. The left panel in Fig 11 shows the sense of familial support from older relatives by group, and the order was TG2 > TG1 > CG, where TG2 was significantly larger than CG (*p* < 0.01). Although the sense of support for TG1 tended to be larger than that for CG, the difference was not statistically significant. Respondents were also asked how much they felt that the IT innovations would support their younger relatives, including their children and grandchildren, on reading the designed messages. The right panel in Fig 11 shows the perception by group that the younger relatives are being supported and was in the order TG2 > TG1 > CG, which was the same as the sense of familial support by older relatives, although the differences were not statistically significant among all the three groups. The larger increase in the perceived familial support by older relatives than that in the perceived support of future generations is similar to our previous study on information provision about air pollution [36].

We investigated the correlation of the intervention effects (*D* for Yourself and Future generations) with the sense of familial support on reading the designed messages (Table 7). For the perception that respondents are being supported by older relatives, CG was marginally significant only for Future generations (*p* < 0.1). The correlation coefficients for TG1 were not significant for either Yourself or Future generations, whereas the correlation coefficients for TG2 were significant for both Yourself and Future generations (*p* < 0.001 and *p* < 0.01, respectively), where the coefficients were the largest in the three groups. Aggregating all three groups, the correlation coefficients for Yourself and Future generations were significant (*p* < 0.001 for both). For the perception that younger relatives of the respondents are being supported by the IT innovations, CG was significant for Yourself (*p* < 0.05) and marginally significant for Future generations (*p* < 0.1). For TG1, the correlation coefficients were significant both for Yourself and Future generations (*p* < 0.001 and *p* < 0.01, respectively). For TG2, the correlation coefficients were significant both for Yourself and Future generations (*p* < 0.001 for both) and were the largest of the three groups. Aggregating all three groups, the correlation coefficients for Yourself and Future generations were significant (*p* < 0.001 for both). All these correlation coefficients were larger than for the perception that respondents are being supported by older relatives. The results of all the correlation analysis suggest that the perception of being supported by older relatives and the perception that younger relatives are being supported by IT innovations could increase positive attitudes.

**Table 7:** Correlation coefficients between change in attitude (*D*) and sense of familial support.

Group	Perception that respondents are being supported by older relatives		Perception that younger relatives of the respondents are being supported by IT innovations	
	Yourself	Future generations	Yourself	Future generations
**CG + TG1 + TG2 (*n* = 3,242)**	***0.06	***0.07	***0.11	***0.09
**CG (*n* = 1,074)**	0.04	‘0.05	*0.06	‘0.05
**TG1 (*n* = 1,091)**	0.03	0.01	***0.11	**0.08
**TG2 (*n* = 1,077)**	***0.11	**0.11	***0.15	***0.13

‘, *, **, *** difference from zero with 90%, 95%, 99%, and 99.9% confidence, respectively.

### Intervention effects by segment

**Fig 12:**
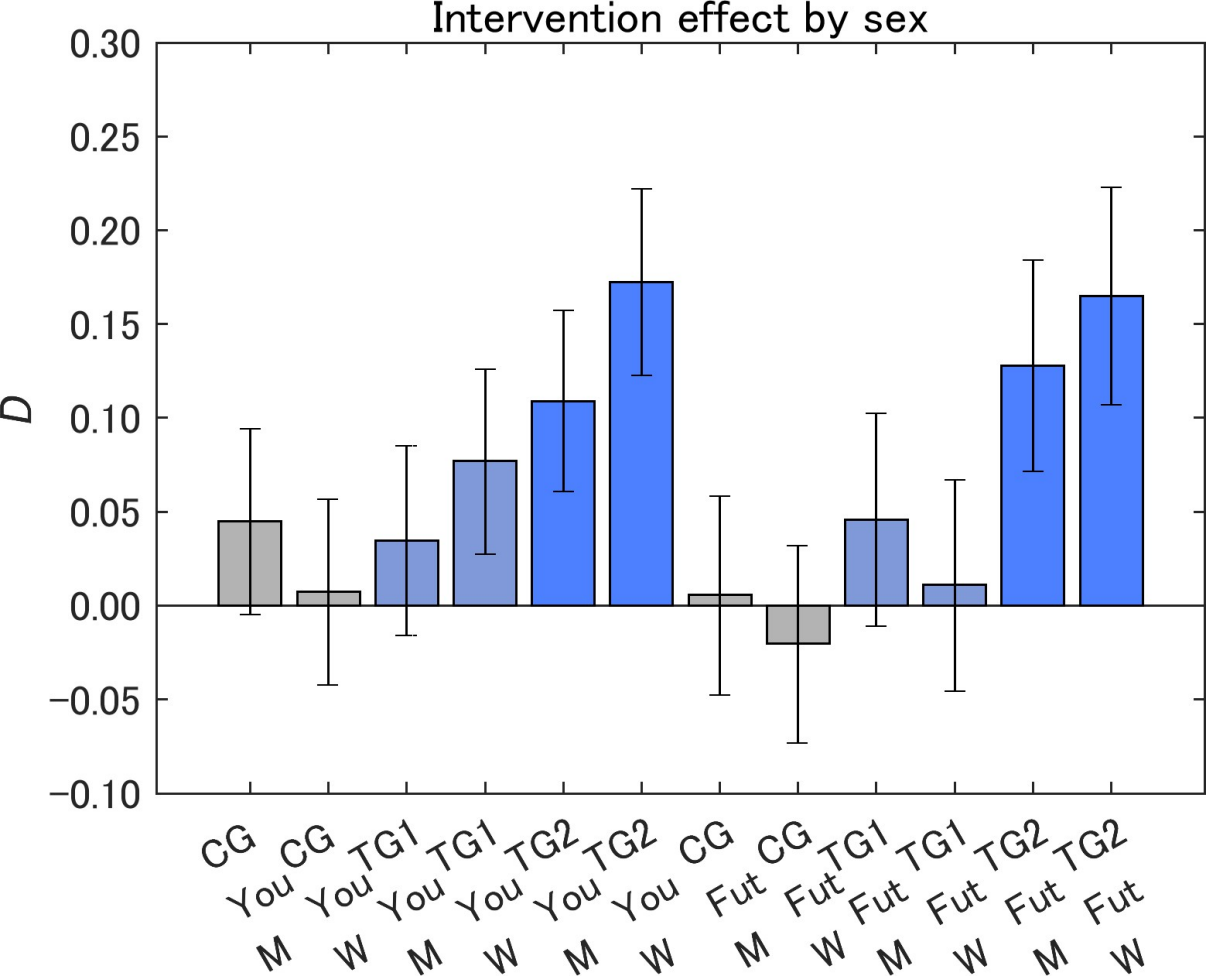
Attitude change toward IT innovations after receiving a designed message (*D*) by sex. The value on the vertical axis is higher the less dangerous the perception of IT innovations became. M, men; W, women. Error bars show 95% confidence intervals.

The samples were subdivided by sex to investigate sex differences in the message effects (Fig 12). The women in TG2 for Yourself showed a marginally significant increase compared with the men (*p* < 0.1), whereas in the other groups there were no other significant differences. The message showed the largest effects on women in TG2. Within the same sex group, the values of *D* were in the order TG2 > TG1 > CG, where the only exception was the men for Yourself. This trend for *D* suggests that the sizes of message effects are TG2 > TG1 > CG independent of sex, which could be reflected in the aggregated results of the same order for both Yourself and Future generations (Fig 10).

In our previous experiment on information provision for air pollution, women showed a significantly larger message effect [36], whereas information provision about IT innovations showed smaller sex differences in the message effects.

**Fig 13:**
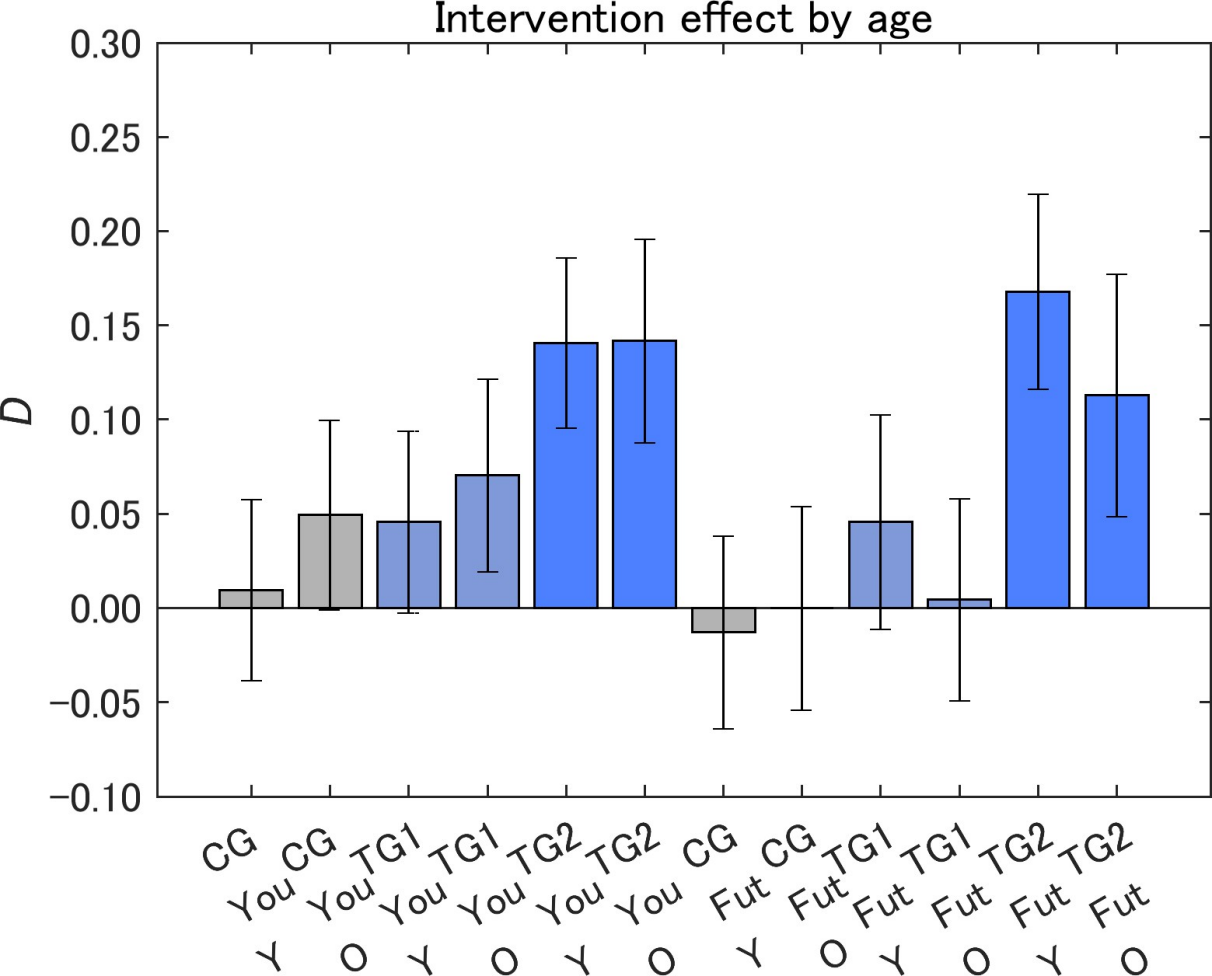
Attitude change toward IT innovations after receiving a designed message (*D*) by age. The value on the vertical axis is higher the less dangerous the perception of IT innovations became. Y, respondents under 50 years old; O, respondents over 50 years old. Error bars show 95% confidence intervals.

The samples were subdivided into groups of younger and older respondents (Fig 13). We defined the younger respondents as under 50 years old and the older respondents as over 50 years old because the message design for TG2 was different for respondents over and under 50 years old (Figs 5 and 6).

In our previous experiment on information provision about air pollution, younger respondents showed a larger significant increase in the message effect [36]; however, in the present study, we found no significant differences in the size of the message effect by age in all groups. The values of *D* were in the order TG2 > TG1 > CG within the same age group, similar to the results for sex. This suggests that the sizes of message effect were in the order TG2 > TG1 > CG independent of whether the respondents were over 50 years old. This was reflected in the aggregated results having the same order for both Yourself and Future generations (Fig 10).

### Panel data analysis

To quantify the DID effects of TG2 messages, we devised the two linear regression models in Eqs (1) and (2) considering sample attributes and personality traits. The personality attributes were sampled using the short Japanese version of the HEXACO model [41, 42], which is an extended version of Big Five model [43].


$$D_{y}=a_{1}\times TG1+a_{2}\times TG2+a_{3}\times S+a_{4}\times A+a_{5}\times P_{hh}+a_{6}\times P_{em\_f}+a_{7}\times P_{em\_a}+a_{8}\times P_{em\_d}+a_{9}\times P_{em\_s}+a_{10}\times P_{ex}+a_{11}\times P_{ag}+a_{12}\times P_{co}+a_{13}\times P_{op}+a_{14}$$ (1)

$$D_{f}=a_{1}\times TG1+a_{2}\times TG2+a_{3}\times S+a_{4}\times A+a_{5}\times P_{hh}+a_{6}\times P_{em\_f}+a_{7}\times P_{em\_a}+a_{8}\times P_{em\_d}+a_{9}\times P_{em\_s}+a_{10}\times P_{ex}+a_{11}\times P_{ag}+a_{12}\times P_{co}+a_{13}\times P_{op}+a_{14}$$ (2)

*TG*1, *TG*2: Target of the intervention in TG1 and TG2, respectively (0: no, 1: yes)

*S*: Sex (0: men; 1: women)

*A*: Age

*P*_*hh*_: Honesty-Humility

*P*_*em_f*_: Fearfulness (Emotionality)

*P*_*em_a*_: Anxiety (Emotionality)

*P*_*em_d*_: Dependence (Emotionality)

*P*_*em_s*_: Sentimentality (Emotionality)

*P*_*ex*_: Extraversion

*P*_*ag*_: Agreeableness

*P*_*co*_: Conscientiousness

*P*_*op*_: Openness

*a*_*1*_–*a*_*13*_: Coefficients for each term

*a*_*14*_: Intercept

We defined *D*_*y*_ as *D* for Yourself and *D*_*f*_ as *D* for Future generations. For HEXACO personality variables, we used four facets only for Emotionality because we identified that respondents with higher Fearfulness contributed significantly more to higher *D*_*y*_ and *D*_*f*_ in our preliminary analysis, whereas Emotionality, which is aggregated from the four facets, showed no significant contribution to the two explained variables. Coefficients *a*_*1*_–*a*_*13*_ and intercept *a*_*14*_ were determined using forced entry regression (Table 8). *TG*2 for Yourself was estimated as 0.114 (*p* < 0.001) and *TG*2 for Future generations, which was our main target variable, was estimated as 0.152 (*p* < 0.001), suggesting that our designed message for TG2 increased positive attitudes toward IT innovations as we intended. The estimates for TG1 were not statistically significant, yet both were positive. The results suggest that the intervention for TG2 with the additional text and illustrations was the largest in the three message groups. For sex and age, the estimates were not significant except for age for Yourself, which was marginally significant (*p* < 0.1) and the value was small, suggesting that these attributes did not have a linear contribution to either explained variable. For the personality variables, Fearfulness, which is a facet of Emotionality, contributed significantly to both explained variables and the values were positive. This suggests that respondents who were sensitive about personal risks were more easily affected by the information provision. Agreeableness was significantly positive for Future generations too, which was consistent with the previous survey for air pollution [36]. Conscientiousness showed a positive contribution only for Yourself, but the effect was weak and marginal (*p* < 0.1).

**Table 8:** Coefficients from linear regression analysis.

Explained variables			Yourself			Future generations		
			Estimated coefficients	SE	*t*	Estimated coefficients	SE	*t*
**Intercept**			***−0.532	0.153	−3.489	−0.247	0.171	−1.443
**Intervention**	TG1		0.030	0.025	1.172	0.036	0.028	1.263
	TG2		***0.114	0.025	4.535	***0.152	0.028	5.375
**Attribute variables**	Sex (Men = 0, Women = 1)		0.007	0.023	0.296	−0.031	0.026	−1.183
	Age		‘0.002	0.001	1.827	−0.001	0.001	−1.307
**Personality variables**	Honesty-Humility		0.022	0.021	1.069	0.023	0.023	1.002
	Emotionality	Fearfulness	**0.054	0.017	3.117	*0.049	0.019	2.537
		Anxiety	−0.001	0.013	−0.069	−0.005	0.015	−0.342
		Dependence	0.002	0.016	0.127	−0.020	0.018	−1.073
		Sentimentality	0.021	0.018	1.180	−0.011	0.020	−0.573
	Extraversion		−0.016	0.021	−0.767	−0.015	0.023	−0.654
	Agreeableness		0.028	0.021	1.309	0.057	*0.024	2.374
	Conscientiousness		‘0.036	0.021	1.702	0.027	0.024	1.118
	Openness		−0.004	0.017	−0.243	0.000	0.019	−0.019
**Adjusted R-squared**			0.012			0.011		
**Number of valid samples**			3242			3242		

‘, *, **, *** difference from zero with 90%, 95%, 99%, and 99.9% confidence, respectively.

### Qualitative survey

The final section of the questions included an open-ended question to ascertain the general impression about the designed messages they read. Although many of the responses were neutral, such as “Nothing special.”, “I have no comment.’, or “I have no idea.”, both positive and negative responses were included. This section presents the typical responses for each message group or attribute of respondents, to extract how the quantitative responses that we explored in the previous section have qualitative meanings.

For CG, a notable neutral response mentioning both the benefits and risks of IT innovations was “I felt anxious about a drop in employment by introducing robots, etc. In the meantime, it seems probable that things human cannot achieve, such as improvement of efficiency or higher quality of products, will be realized.” from a 24-year-old woman, who did not change her attitudes toward IT innovations. This kind of response suggests that some people recognize the trade-off between the benefits and the risks, which results in their attitudes being unchanged. A positive response was “We should rely on robots for tasks that can be done by automation because manpower is scarce.” from a 51-year-old man who changed his attitudes to safer by 1 point for both Yourself and Future generations. Another positive response was “Although I had an image that employment will be taken over by robots, care-giving robots, etc. will be required due to the growing number of elderly people.” from a 48-year-old woman who changed her attitudes to safer by 1 point for both Yourself and Future generations. These positive responses may be based on the idea that robots are useful to complement human resources in some specific areas where the scarcity of manpower is becoming evident already, rather than thinking that robots will take over existing jobs. Typical negative responses were about the job take-over, for example “Although IT innovations have made our everyday life more convenient, there are many aspects that have become less convenient. There are many potential problems, such as decreased employment in the future as a result of robots taking over jobs, or elderly people who cannot keep up with the changes in technology.” from a 67-year-old woman who changed her attitude to more dangerous by 1 point for Future generation. Another negative response was about excessive dependence on technologies, such as “Although I think IT innovations have made everyday life more convenient and will help to complement work and labor where manpower is scarce, I feel more anxious about situations where we cannot connect to the Internet or cannot use electricity, in the meantime.” from a 46-year-old woman who changed her attitudes to more dangerous by 1 point for both Yourself and Future generations.

A notable response for TG1 was “I could not imagine a concrete situation and I felt it was difficult to understand.” from a 61-year-old man who did not change his attitudes. A similar response was “It did not appeal to me so much and it was difficult to read.” from a 25-year-old woman who did not change her attitudes. This kind of response suggesting that respondents were reluctant to read through the text was not observed from the people in CG, who were presented with shorter messages. In our previous survey, we observed that some people felt that information comprising only text with additional sentences emphasizing familial support was lengthy, even if the sentences were short [36]. This tendency was observed again in the present survey.

Responses that characterize TG2 mentioned familial support, which increased their positive attitudes toward the IT innovations. One example was “Although I was wondering so much what IT was and why IT jobs were profitable more than 10 years ago, now I am thinking that IT is essential because many parts of modern daily life directly receive its benefits.” from a 34-year-old woman who changed her attitude to safer by 1 point for Yourself. Another similar response was “I thought that each generation has been influenced by the previous generation and passes their influence on to the next generation.” from a 67-year-old woman who changed her attitudes to safer by 1 point for both Yourself and Future generations. TG2 still had similar neutral and negative responses to those observed in the other two message groups. An example of a neutral response was “Although I feel anxious about employment because there will be more cases where humans are not needed, IT seems fairly beneficial, considering population decrease.” from a 43-year-old man who did not change his attitudes for Yourself or Future generations. A negative response was “I think society risks becoming a movie-like world (he mentioned a famous film name) where humans are taken over by machines.” from a 43-year-old man who changed his attitude to more dangerous by 1 point for Future generations. Another negative response was that “I feel anxious about job losses despite the convenience. I guess we will have no way to earn money.” from a 53-year-old woman who changed her attitudes to safer by 1 point for both Yourself and Future generations.

We also focused on the relationships between the intervention effects and the two personality traits that contributed significantly to the intervention effect in the panel analysis (Table 8). First, samples with high *D* for Future generations and high Fearfulness were extracted. A characteristic response from the extracted samples was “Dangerous tasks can be avoided, and an extraordinary number of products can be manufactured, which cannot be achieved by manpower, thanks to IT.” from a 44-year-old woman with a Fearfulness score of 4.67 (value range is 1–5, mean value is 3.48, and standard deviation is 0.71) who changed her attitudes by 1 point for both Yourself and Future generations. Respondents with high Fearfulness may expect that automation could be used for tasks that are risky for humans, and thus they may interpret the information as suggesting a decrease in risk. Another typical response from this segment showed that they expected traffic accidents to be decreased by self-driving cars and improvements in medical technologies. One such example was “I think it is good that IT innovations improve medical technologies and enable difficult surgeries. I want it to benefit not only a limited wealthy class but also the general public. I also want it to prevent traffic accidents caused by human error. Large trucks that avoid hitting pedestrians on turning and technologies enabling the cooperation of cars and traffic signals are beneficial too.” from a 46-year-old woman with a Fearfulness score of 5.0 who changed her attitudes by 1 point for both Yourself and Future generations. Second, we extracted samples with high *D* for Future generations and high agreeableness. A characteristic response was “I think IT will make more things more convenient and helpful. I am glad about that and want IT to be rigorously sophisticated as long as it is useful for everyone. At the same time, I think that everything comes with risks, which I want to be transparent. However convenient it will become, I want to value interpersonal connections.” from a 45-year-old woman with an Agreeableness score of 3.8 (value range is 1–5, mean value is 2.93, and standard deviation is 0.51) who changed her attitudes by 1 point for both Yourself and Future generations. This suggests that respondents with high agreeableness tend to expect that IT should benefit more people in a humane way, and thus they accept the information more readily.

## Discussion

The present study investigated the effects of our designed messages reminding people of familial support to increase positive attitudes toward IT innovations. The messages showed statistically significant effects, similar to our previous study of nudging attitudes toward air pollution caused by industrialization [36].

There were three main similarities to our previous study. First, the effect sizes were in the order CG (the simplest textual message) < TG1 (textual message with additional descriptions of familial support) < TG2 (textual message with additional descriptions of familial support and an illustration), where the size of the effect was strongly correlated with perceptions of being supported by previous generations. Adding a textual description of support from previous generations and to future generations in TG1 increased perceptions of familial support and the effects of information provision to increase positive attitudes toward IT innovations. Further additional illustrations increased the perceptions of familial support and the effect of information provision, even though the illustration contained the same information as the textual descriptions for TG1. Second, before the intervention, the respondents initially perceived the influence of the risk sources as larger on Future generations than on Yourself, and women were more risk-averse. Third, respondents with high agreeableness showed larger intervention effects on Future generations, where they expected the IT innovations to benefit more people.

These consistent trends in the effects suggest that the framework of our designed message could be applied to information provision for a wider variety of topics. The message effects of increasing positive attitudes toward new technologies are especially meaningful. Introducing new technologies to society is difficult irrespective of the quantitative risks or benefits, due to the low familiarity of the technologies to the general public, which is associated with risk-averse attitudes. The proposed framework of information provision could be used to promote new technologies other than IT technologies.

However, there were also several differences from the previous study. First, the message effect of TG2 for Yourself, not just for Future generations, was much stronger and statistically significant compared with CG. This may be because IT is a current hot topic that affects respondents directly via personal computers or smartphones, whereas the air pollution caused by industrialization investigated in the previous study is much improved and they rarely see relevant information in their daily life, even though they perceive the risks of the air pollution as much higher than those of IT innovations [36]. Second, age showed a weak positive contribution to the intervention effects, whereas the contribution was negative in the previous study. The cause of this discrepancy is unclear, but one possible interpretation could be that older respondents might be expecting benefits related to familiar events in daily life; for example, they may visit hospitals more often and have more difficulty driving. Another possible interpretation could be a bias caused by the survey method. Although older people are generally resistant to new technology [47], they did not appear to be resistant to the IT innovations before receiving the designed messages in the present study, where the numbers of old respondents were limited, and thus the tendency was not significant. The present survey used internet-based questionnaires, for which the respondents are required to use personal computers or smartphones. This could select more respondents who are originally open to IT technology and include older respondents who are less resistant to IT innovations. Third, unique to the present study was that respondents with high Fearfulness, which is a facet of Emotionality, showed larger intervention effects for both Future generations and Yourself. This result can be explained by the expectation that IT innovations will help to reduce risks in everyday life in the future. They mentioned diseases and traffic accidents as risks that they hope will be improved, instead of the risks of job loss.

These kinds of responses showing expectations of realistic benefits from AI are consistent with previous studies on public perception of AI risks. For example, Stephen Hawking’s warning about how we should handle AI technologies [48] greatly influenced the risk perception of AI by the general public. Data analysis using textual data posted on social media suggested that his influence has amplified the public perception of the existential risk that AI will evolve autonomously and threaten the existence of human beings in the future, as in certain science fiction films. However, fatal accidents that have already occurred, such as test-driving failures in self-driving cars, are a minor concern to the general public and have not contributed to an increase in the risk perception [49]. Likewise, responses mentioning actual accidents related to AI were minor in our study too. Conversely, the expectation of the future benefits appeared to increase the intervention effects of our designed messages.

Regarding the differences in personality effects on the pre-intervention attitudes and the attitude change, the relationship between the pre-intervention risk-averse attitudes and HEXACO measures (Table 6) was generally consistent with previous studies [50, 51]. Honesty-Humility and Emotionality were positively correlated and Extraversion, Agreeableness, Conscientiousness, and Openness were negatively correlated with the pre-intervention risk-averse attitudes, and all the coefficients were significant. In the meantime, these tendencies were not observed in the attitude change caused by the designed messages (Table 8). Thus, how the message effects are affected by personality traits is separate from the attitudes in normal conditions. The designed messages appear to stimulate some specific personality traits, as seen in the positive contribution of Fearfulness to the message effects for both Future generations and Yourself, or Altruism for Future generations.

One of the most influential models for understanding technology acceptance, TAM [39, 40] and its variations, considers perceived usefulness and perceived ease-of-use with a variety of additional factors, such as subjective norms [52–55] and trust in influencers [56]. Considering the criticism that a series of TAM models can explain only about 40% of the variance in using technology [57], the message effects of promoting a sense of familial support investigated in our study may explain some of the rest of the unidentified factors.

The boosted message effect of providing additional visual information with textual information in the present study may correspond to the multimedia effect [58, 59], which is the observation that a combination of visual and textual information promotes better learning than either type of information alone [60, 61]. A combination of textual and visual information or only visual information, without highlighting familial support, may have message effects, the sizes of which are unknown. These non-familial messages and our designed messages should be compared in future studies.

The balance of positive and negative sentences in the designed messages may also have mattered; the first two positive sentences were longer than the last single negative sentence, even in the CG. The message effect for the CG was not significantly different from 0 (Fig 10), which could be interpreted as indicating that the message effect was ideally neutral. Meanwhile, the positive sentences and the negative sentence might have opposite message effects, and the neutral message effect might occur because of the combined effect of the sentences. Evaluating these positive and negative message effects by subdividing the message group further would help to understand more individual effects.

## Conclusion

We performed an RCT to investigate using designed messages to increase positive attitudes toward IT innovations. Before receiving the designed messages, the respondents perceived the IT innovations to threaten future generations more than the respondents themselves. The message effects were statistically significant; that is, the positive attitudes of respondents who perceived stronger support from previous generations on reading the messages were increased more. The sense of familial support and the intervention effects were boosted by viewing an illustration showing the relationship of how previous generations benefit present and future generations via IT innovations, compared with only reading a textual message containing the same information. The intervention effects were also statistically significant for the perceived risks to the respondents themselves, not just to future generations.

We analyzed which personality traits contributed to the message effects using the HEXACO model. Fearfulness, which is a facet of Emotionality, boosted the intervention effects on the perceived risks to both the respondents themselves and to future generations. Responses to an open-ended question suggested that respondents with high Fearfulness were expecting IT innovations to help to reduce risks in daily life, such as improving medical treatments or preventing traffic accidents. Agreeableness boosted the intervention effects too, but only for perceived risks to future generations. Typical responses were expectations that IT innovations should benefit the general public.

Our previous study showed that a designed message had similar effects on attitudes to risk from air pollution caused by industrialization to our designed message about IT innovations [36]. Although there were several different responses from the previous study by segment on receiving our designed messages, we demonstrated that our framework could be used for a wide variety of applications involving information provision perceived to involve future generations.

Examples of further case studies for potential applications are low-carbon technologies or carbon recycling technologies, which would have a large influence on future society. In particular, technologies that will need large infrastructure will encounter not-in-my-back-yard problems, which describe the phenomenon where new public assets are approved of by the general public but are not accepted by residents of the place where the assets are built. These changes may encounter risk-averse attitudes from local residents, even if the social benefits are clear. Thus, we will investigate how our designed message framework can be used for information provision about these new technologies in future work. Our two case studies do not guarantee that our framework is universally effective for information provision about every kind of risk because it is natural that there are limitations to the applicability of the method, and our future work will also explore under which conditions the designed messages are effective or can have negative effects.

## Supporting information

S1 File (ZIP)
