# Supplementary material for: Nudging attitudes toward IT innovations by information provision that serves as a reminder of familial support
Source: PLoS One. 2023 Feb 24;18(2):e0282077. doi: 10.1371/journal.pone.0282077 (PMC9955986; doi:10.1371/journal.pone.0282077)
Supplement: S1 File — (ZIP) [file pone.0282077.s001.zip › S1_and_S2.docx]

**S1 Questionnaire**

**Questions for interventions (in English)**

***Q*_pre_**

How do you think IT innovations affect the following people in daily life?

|  | **Safe** | **Slightly safe** | **Neutral** | **Slightly dangerous** | **Dangerous** |
| --- | --- | --- | --- | --- | --- |
| **Future generations** | 1 | 2 | 3 | 4 | 5 |
| **Yourself** | 1 | 2 | 3 | 4 | 5 |

***Q*_post_**

(One of the messages for CG, TG1, and TG2 is shown.**)**

**[Please read the message about IT innovations and answer the question.]**

Please answer the question below again. How do you think IT innovations affect the following people in daily life?

|  | **Safe** | **Slightly safe** | **Neutral** | **Slightly dangerous** | **Dangerous** |
| --- | --- | --- | --- | --- | --- |
| **Future generations** | 1 | 2 | 3 | 4 | 5 |
| **Yourself** | 1 | 2 | 3 | 4 | 5 |

**S1 Table. Summary Statistics for *Q*_pre_ and *Q*_post_.**

| **Question** | | **Mean** | **Standard deviation** | **Min** | **Max** |
| --- | --- | --- | --- | --- | --- |
| ***Q*_pre_** | **Future generations** | 2.77 | 0.89 | 1 | 5 |
|  | **Yourself** | 2.68 | 0.79 | 1 | 5 |
| ***Q*_post_** | **Future generations** | 2.71 | 0.92 | 1 | 5 |
|  | **Yourself** | 2.60 | 0.81 | 1 | 5 |

**Other questions (in English)**

***Q*1**

(One of the messages for CG, TG1, and TG2 is shown.**)**

Upon reading the message above, do you think you are receiving benefits that increase your health and quality of everyday life from your older relatives, including parents or grandparents?

| **I am benefitting** | **I am benefitting slightly** | **I am not benefitting much** | **I am not**  **benefitting** |
| --- | --- | --- | --- |
| 1 | 2 | 3 | 4 |

***Q*2**

(One of the messages for CG, TG1, and TG2 is shown.**)**

Upon reading the message above, do you think IT innovations is giving benefits of increasing health and quality of everyday life to your younger relatives, including children or grandchildren?

| **It has benefits** | **It has some benefits** | **It has few benefits** | **It does not**  **have benefits** |
| --- | --- | --- | --- |
| 1 | 2 | 3 | 4 |

***Q*3**

(One of the messages for CG, TG1, and TG2 is shown.**)**

Please let us know your impressions or thoughts when you read the above message.

***Q*4**

Please answer each of the questions below.

#Please answer about your children.

#Please answer 0 for all the questions if you have no children now.

1. How many children do you have?
2. How many children are living with you?
3. How many children are working in a paid job?

***Q*5**

Please let us know your parents’ current status about their living and working. (Please check as many as necessary for each question.)

|  | **Father** | **Mother** | **None** |
| --- | --- | --- | --- |
| 1. **Living with you in the same house (or at the same site)** |  |  |  |
| 1. **Working in a paid job** |  |  |  |

***Q*6**

Do you think you are being “supported” by the following people somehow?

By your parents, grand-parents, sons, or daughters.

| **Supported** | **Supported a little** | **Not supported much** | **Not supported** |
| --- | --- | --- | --- |
| 1 | 2 | 3 | 4 |

**Questions for interventions (in Japanese)**

***Q*_pre_**

ITの技術革新が、普段の生活で次の方に与える影響について、あなたはどう思いますか？

|  | **安全** | **やや安全** | **どちらともいえない** | **やや危険** | **危険** |
| --- | --- | --- | --- | --- | --- |
| **未来の世代に** | 1 | 2 | 3 | 4 | 5 |
| **あなた自身に** | 1 | 2 | 3 | 4 | 5 |

***Q*_post_**

(CG, TG1, TG2いずれかのメッセージを表示)

**【ITの技術革新に関する説明を読んで、お答えください。】**

改めて伺います。ITの技術革新が、普段の生活で次の方に与える影響について、あなたはどう思いますか？

|  | **安全** | **やや安全** | **どちらともいえない** | **やや危険** | **危険** |
| --- | --- | --- | --- | --- | --- |
| **未来の世代に** | 1 | 2 | 3 | 4 | 5 |
| **あなた自身に** | 1 | 2 | 3 | 4 | 5 |

**Other questions (in Japanese)**

***Q*1**

(CG, TG1, TG2いずれかのメッセージを表示)

上記の内容を読んで、あなたは次の方から健康・日常生活の質向上などの恩恵を受けていると思いますか？

ご両親やご祖父母など、ご自分より年上の血縁者から

| **受けている** | **やや受けている** | **あまり受けていない** | **受けていない** |
| --- | --- | --- | --- |
| 1 | 2 | 3 | 4 |

***Q*2**

(CG, TG1, TG2いずれかのメッセージを表示)

上記の内容を読んで、ITの技術革新が次の方に対して健康・日常生活の質向上などの恩恵を与えると思いますか？

あなたのお子さんやお孫さんなど、ご自分より年下の血縁者の方へ

| **与える** | **やや与える** | **あまり与えない** | **与えない** |
| --- | --- | --- | --- |
| 1 | 2 | 3 | 4 |

***Q*3**

(CG, TG1, TG2いずれかのメッセージを表示)

上記の内容を読んでの印象・ご感想をお聞かせください。

***Q*4**

以下の項目についてそれぞれお答えください。

※あなたご自身の、血縁関係にあるお子さんについてお答えください。

※現在お子さんがいない方は全て0とお答えください。

1. 現在お子さんは何人いらっしゃいますか？
2. 現在同居されているお子さんは何人いらっしゃいますか？
3. 現在収入のある仕事をされているお子さんは何人いらっしゃいますか？

***Q*5**

現在のあなたご自身の親御さんとの同居状況と、親御さんの仕事の状況についてお知らせください。（それぞれいくつでも）

|  | **父** | **母** | **あてはまる人はいない** |
| --- | --- | --- | --- |
| 1. **現在同居している（または同じ敷地内に同居）** |  |  |  |
| 1. **現在収入のある仕事をしている** |  |  |  |

***Q*6**

あなたは次の方から、有形無形を問わず、普段何らかのかたちで「支援を受けている」と思いますか？

ご両親やご祖父母、娘・息子から

| **受けている** | **やや受けている** | **あまり受けていない** | **受けていない** |
| --- | --- | --- | --- |
| 1 | 2 | 3 | 4 |

**S2 Appendix**

**Tables**

Table A shows the numbers of samples by region. The sample ratios were similar to the census data, reflecting the random collection of samples independent of the region.

**Table A. Sample Ratios by Region.**

| **Region** | **Collected samples** | | **Percentage from census (%)^a^** |
| --- | --- | --- | --- |
|  | **Percentage (%)** | **Counts** |  |
| **Hokkaido** | 5.2 | 168 | 4.7 |
| **Tohoku** | 5.3 | 172 | 6.6 |
| **Kanto** | 37.4 | 1211 | 36.8 |
| **Chubu** | 16.9 | 548 | 15.0 |
| **Kinki** | 19.0 | 615 | 16.5 |
| **Chugoku** | 5.3 | 172 | 5.9 |
| **Shikoku** | 2.6 | 85 | 3.1 |
| **Kyushu** | 7.6 | 247 | 10.4 |
| **Okinawa** | 0.7 | 24 | 1.1 |

^a^Retrieved from the 2019 data at https://www.stat.go.jp/data/kakei/setai_bunpu.html.

Regions are listed from north to south.

**Figures**

Figs A–C show the number of children, children living with the respondents, and children who are working, respectively. The mean numbers for each attribute were 1.1, 0.7, and 0.4, respectively.

**Fig A. Number of Respondents’ Children.**

**Fig B. Number of Children Living With Respondent.**

**Fig C. Number of Respondents’ Children Who Are Working.**

Figs D and E show the number of parents who are living with the respondents and respondents who are working, respectively. The mean numbers for each attribute were 0.3 and 0.5, respectively.

**Fig D. Number of Parents Who Are Living With Respondent.**

**Fig E. Number of Respondents’ Parents Who Are Working.**
