# Supplementary figures and images for: Nudging attitudes toward IT innovations by information provision that serves as a reminder of familial support
Source: PLoS One. 2023 Feb 24;18(2):e0282077. doi: 10.1371/journal.pone.0282077 (PMC9955986; doi:10.1371/journal.pone.0282077)

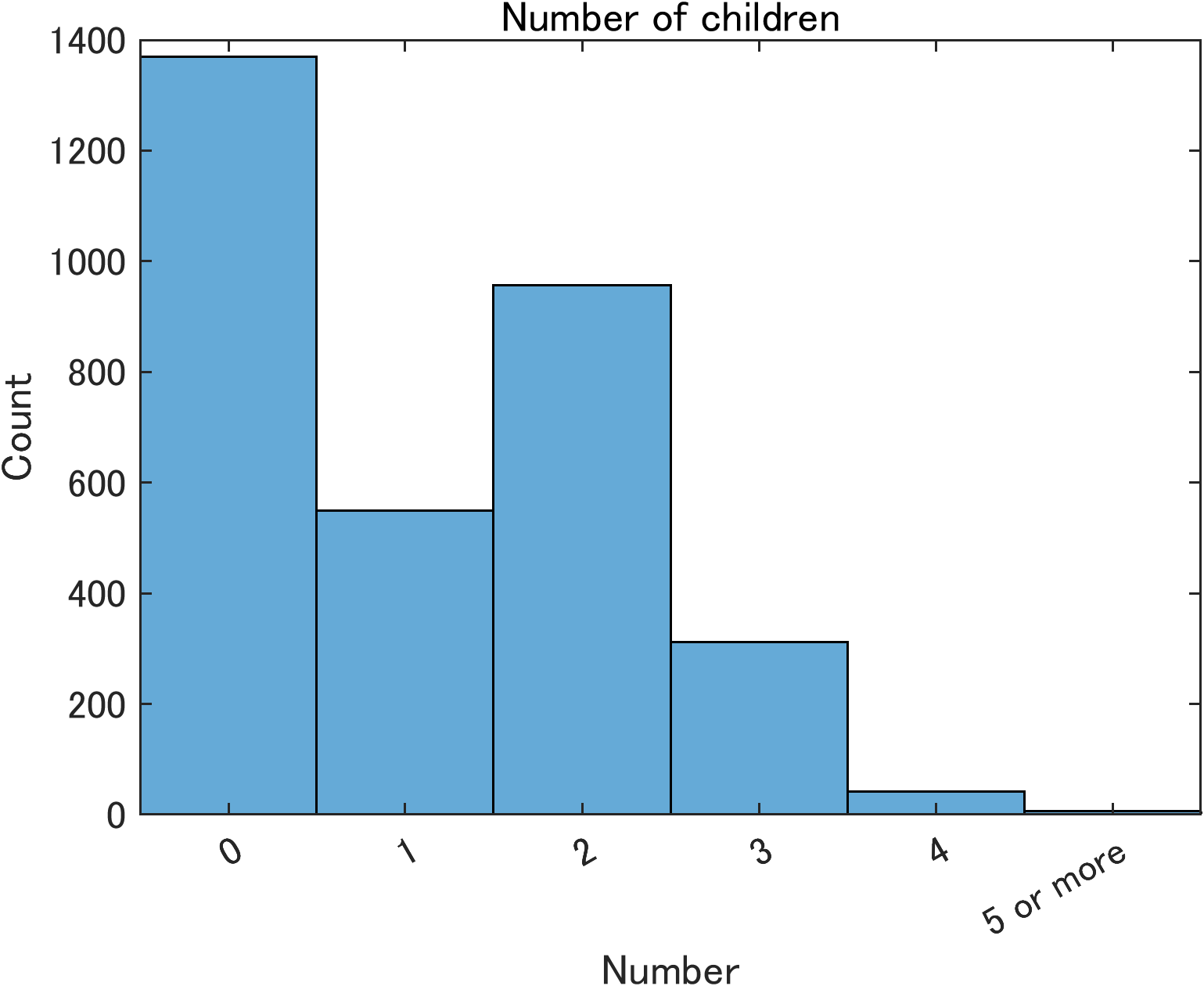

Supplement: S1 File — (ZIP) [file pone.0282077.s001.zip › S2 FigA.tif]

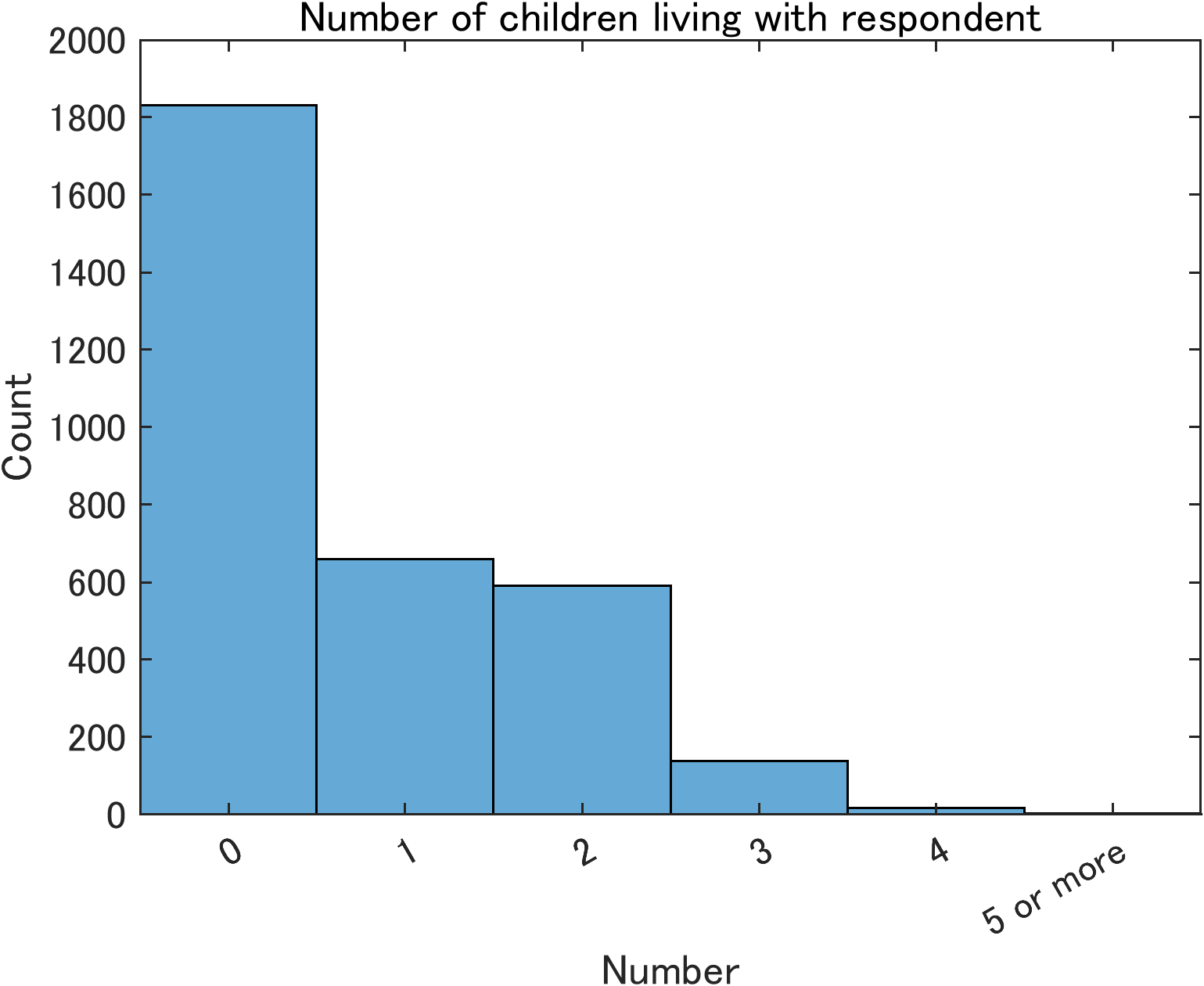

Supplement: S1 File — (ZIP) [file pone.0282077.s001.zip › S2 FigB.tif]

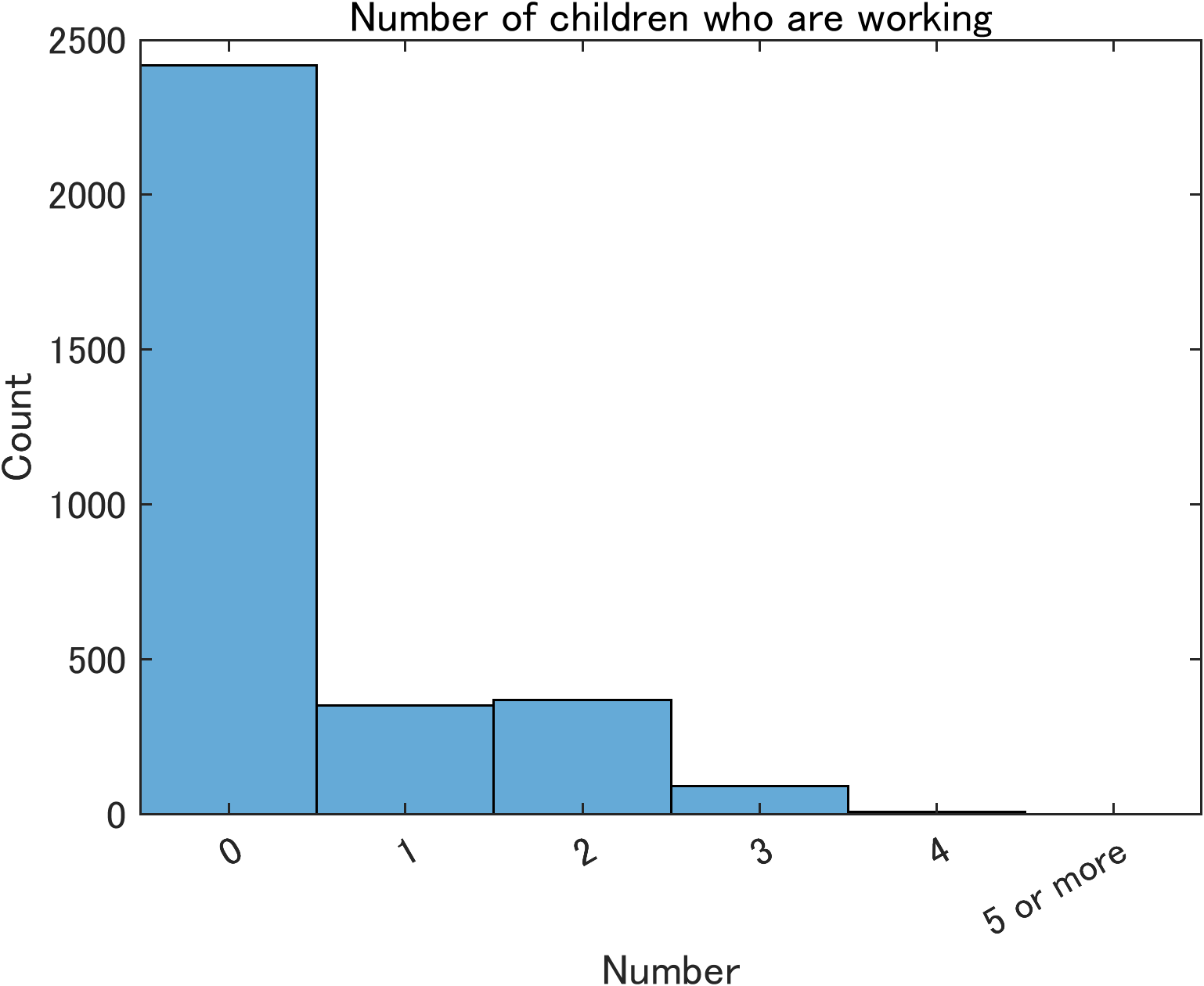

Supplement: S1 File — (ZIP) [file pone.0282077.s001.zip › S2 FigC.tif]

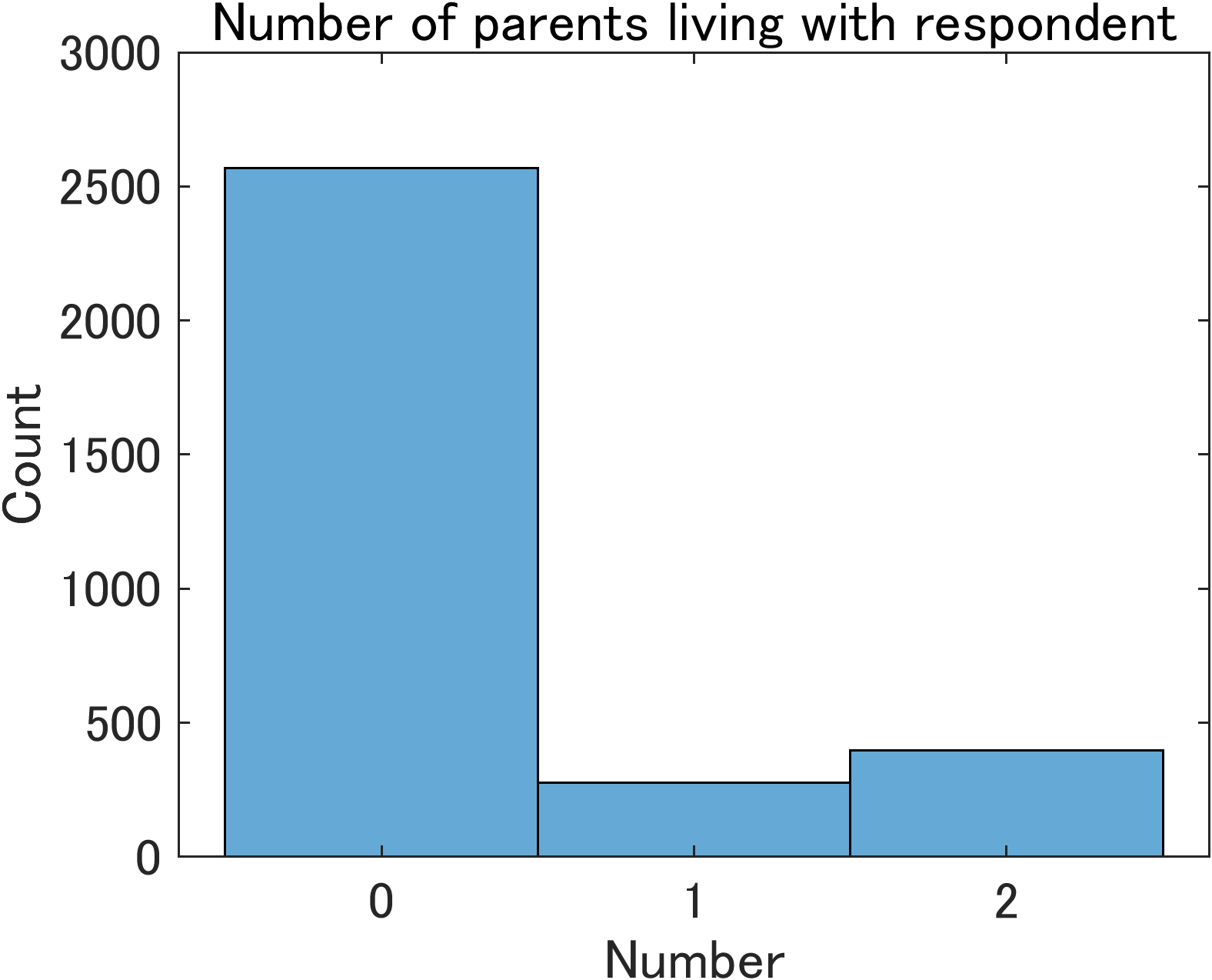

Supplement: S1 File — (ZIP) [file pone.0282077.s001.zip › S2 FigD.tif]

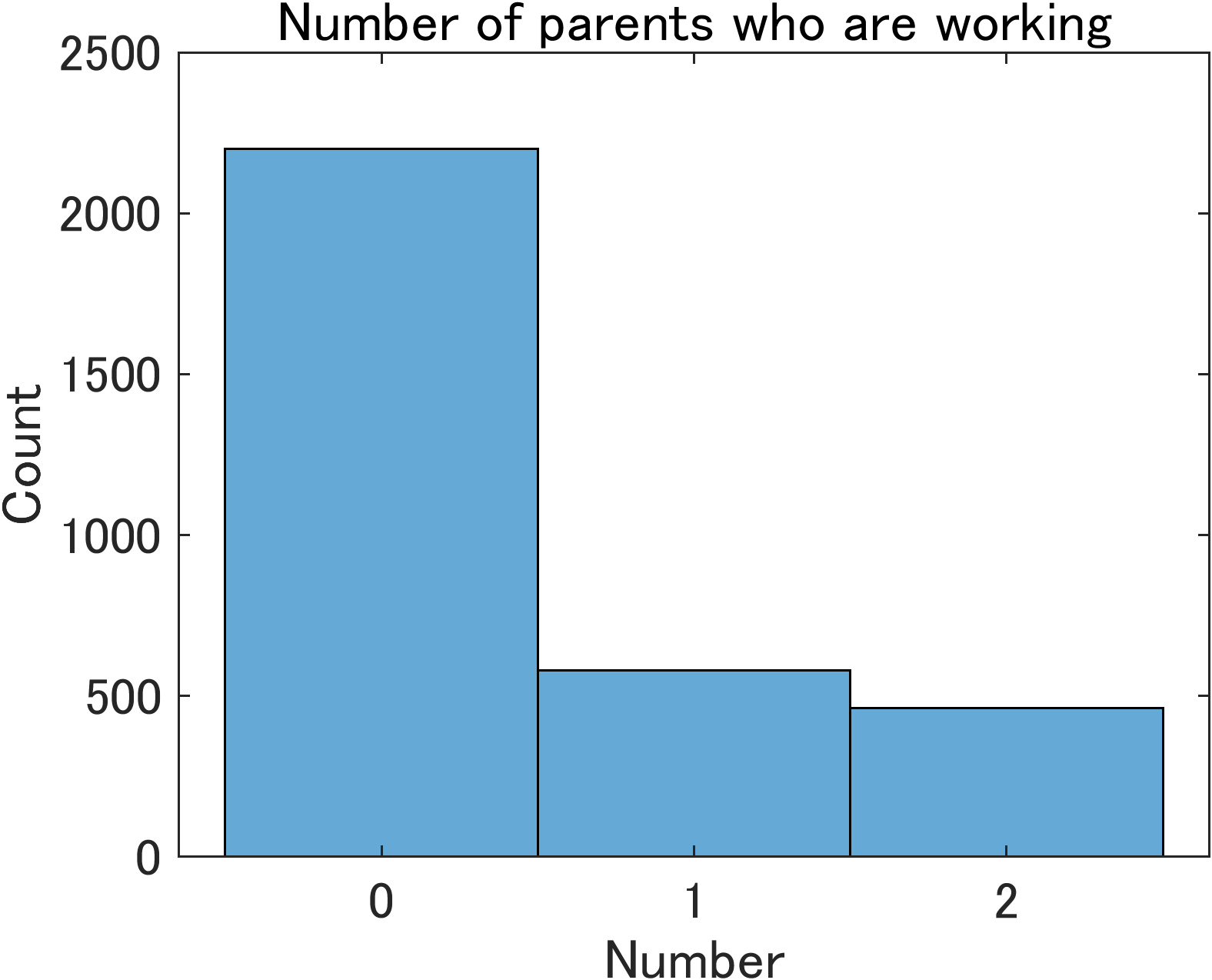

Supplement: S1 File — (ZIP) [file pone.0282077.s001.zip › S2 FigE.tif]
